# Protocol for a randomized study assessing the feasibility of home-based albuminuria screening among the general population: The THOMAS study

**DOI:** 10.1371/journal.pone.0279321

**Published:** 2022-12-22

**Authors:** Dominique van Mil, Lyanne M. Kieneker, Birgitte Evers-Roeten, Marc H. M. Thelen, Hanne de Vries, Marc H. Hemmelder, Annemiek Dorgelo, Ronald W. van Etten, Hiddo J. L. Heerspink, Ron T. Gansevoort

**Affiliations:** 1 Division of Nephrology, Department of Internal Medicine, University Medical Center Groningen, University of Groningen, Groningen, The Netherlands; 2 Department of Clinical Pharmacy and Pharmacology, University Medical Center Groningen, University of Groningen, Groningen, The Netherlands; 3 General Practice Tholos, Zevenbergen, The Netherlands; 4 Result Laboratory and Clinical Chemistry, Amphia Hospital, Breda, The Netherlands; 5 Department of Internal Medicine, Jeroen Bosch Hospital, ’s-Hertogenbosch, The Netherlands; 6 Department of Internal Medicine, CARIM Cardiovascular Research Institute, Maastricht University Medical Center, University Maastricht, Maastricht, The Netherlands; 7 Dutch Kidney Foundation, Bussum, The Netherlands; 8 Department of Internal Medicine, Amphia Hospital, Breda, The Netherlands; Medical College of Wisconsin, UNITED STATES

## Abstract

**Background:**

Chronic kidney disease (CKD) is a rising public health problem that may progress to kidney failure, requiring kidney replacement therapy. It is also associated with an increased incidence of cardiovascular disease (CVD). Because of its asymptomatic nature, CKD is often detected in a late stage. Population screening for albuminuria could allow early detection of people with CKD who may benefit from preventive treatment. In case such screening is performed in a general practitioner (GP) setting, this will result in relatively high costs. Home-based screening might be an effective and cost-effective alternative.

**Aim:**

The THOMAS study (Towards HOMe-based Albuminuria Screening) is designed to prospectively investigate two methods for home-based population screening for increased albuminuria to detect yet undiagnosed CKD and risk factors for progression and CVD.

**Methods:**

This investigator initiated, randomized population-based study will include 15.000 individuals aged 45–80 years, who will be randomly assigned to be invited for a home-based screening test for albuminuria with a more conventional urine collection device or an innovative smartphone application. If the test result is positive upon confirmation (i.e., elevated albuminuria), participants are invited to a central screening facility for an elaborate screening for CKD and CVD risk factors. Participants are referred to their GP for appropriate treatment, if abnormalities are found. Primary endpoints are the participation rate, yield, and cost-effectiveness of the home-based screening and elaborate screening.

**Conclusions:**

The THOMAS study will evaluate the effectiveness and cost-effectiveness of home-based albuminuria screening in the general population for the early detection of CKD and CVD risk factors. It will provide insight into the willingness to participate in population screening for CKD and into the compliance of the general population to a corresponding screening protocol and compliance to participate. Thus, it may help to develop an attractive novel screening strategy for the early detection of CKD.

**Trial registration:**

ClinicalTrials.gov, registration number NCT04295889, registered 05 March 2020. https://www.google.com/search?client=firefox-b-d&q=NCT04295889.

## Introduction

Chronic kidney disease (CKD) is a public health problem with a rising global prevalence that currently exceeds 10% [1,2]. CKD may progress to kidney failure, requiring dialysis or transplantation [3]. Importantly, patients with CKD, even in the earliest stages, are at increased risk for cardiovascular disease (CVD) [4]. These sequelae result in an impaired quality of life, decreased life expectancy, and a high economic burden for society [3]. CKD often remains undiagnosed for a long time and therefore not treated [5]. Therefore, earlier identification of CKD is desired to allow a timely start of treatment to prevent progression and associated complications [6].

Traditionally, most attention to screen for CKD was focused on measuring kidney function. However, screening for impaired kidney function allows only late intervention. During the last two decades, it has become clear that increased albuminuria is a strong risk factor for progressive CKD. In addition, it also predicts CVD as a marker of generalized vascular endothelial damage [7–9]. In the PREVEND study, that was performed in 1997, 5.6% of the general population had confirmed elevated albuminuria (i.e., urinary albumin excretion (UAE) ≥ 30 mg/24h) [10]. In the majority of these subjects, elevated albuminuria was not known to these subjects nor their general practitioners (GP), and 79% of them also had hypertension, type 2 diabetes, or hypercholesterolemia. In 89% of these individuals, one of these comorbidities was not yet diagnosed. Additionally, it was found that screening for increased albuminuria identified patients with a preserved kidney function, but a more pronounced decline in estimated GFR (eGFR) thereafter [8], and a cardiovascular (CV) event rate twice as high as in normoalbuminuric patients with a similar CV risk factor profile [11].

Screening for albuminuria could thus provide an opportunity to detect people with early stage CKD who may benefit from preventive treatment. Therefore, population screening for albuminuria has been proposed. However, the cost-effectiveness of such a screening strategy has been questioned [12–14]. Of note, these screening programs in general were modeled as screening for severely elevated albuminuria (i.e., albumin-to-creatinine ratio (ACR) ≥300 mg/g, corresponding with a 24hr UAE ≥300 mg/24hr), with screening performed by physicians (e.g., GPs), and taking into account only benefit for prevention of kidney failure. When screening is performed this way, it will identify only a few subjects, the costs for screening will be high, and the potential benefits limited. Another screening strategy has therefore been proposed, i.e., a home-based pre-screening for moderately elevated albuminuria (i.e., ACR ≥30 mg/g, corresponding with a 24hr UAE ≥30 mg/24hr), with subsequent screening by GPs of only those with proven elevated albuminuria, and taking into account not only prevention of kidney failure but also benefits with respect to prevention of CVD. Such a screening strategy has been suggested to be cost-effective [15].

In recent years, albuminuria screening tests have become available making home-based population screening for elevated albuminuria feasible. The present paper describes the design and rationale of the Towards HOMe-based Albuminuria Screening (THOMAS) study, a randomized study investigating the feasibility of population screening for increased albuminuria by two different methods.

## Materials and methods

### Study setting and population

The THOMAS study is designed as an investigator-initiated, prospective, randomized population-based screening study. The University Medical Center Groningen, the Netherlands, acts as the coordinating center. Individuals from the general population are eligible if they are aged 45–80 years and living in the region of Breda, the Netherlands. Individuals are excluded when institutionalized. A random sample of men and women aged 45–80 years living in the region of Breda is drawn by Statistics Netherlands. This sample represents the Dutch population with respect to distribution of age, sex and socioeconomic class (as assessed by postal code area) [16]. The Dutch Ministry of the Interior and Kingdom Relations provides names, dates of birth, sex, and addresses.

### Study design and procedures

#### Study preparation, randomization, and recruitment

**Fig 1:**
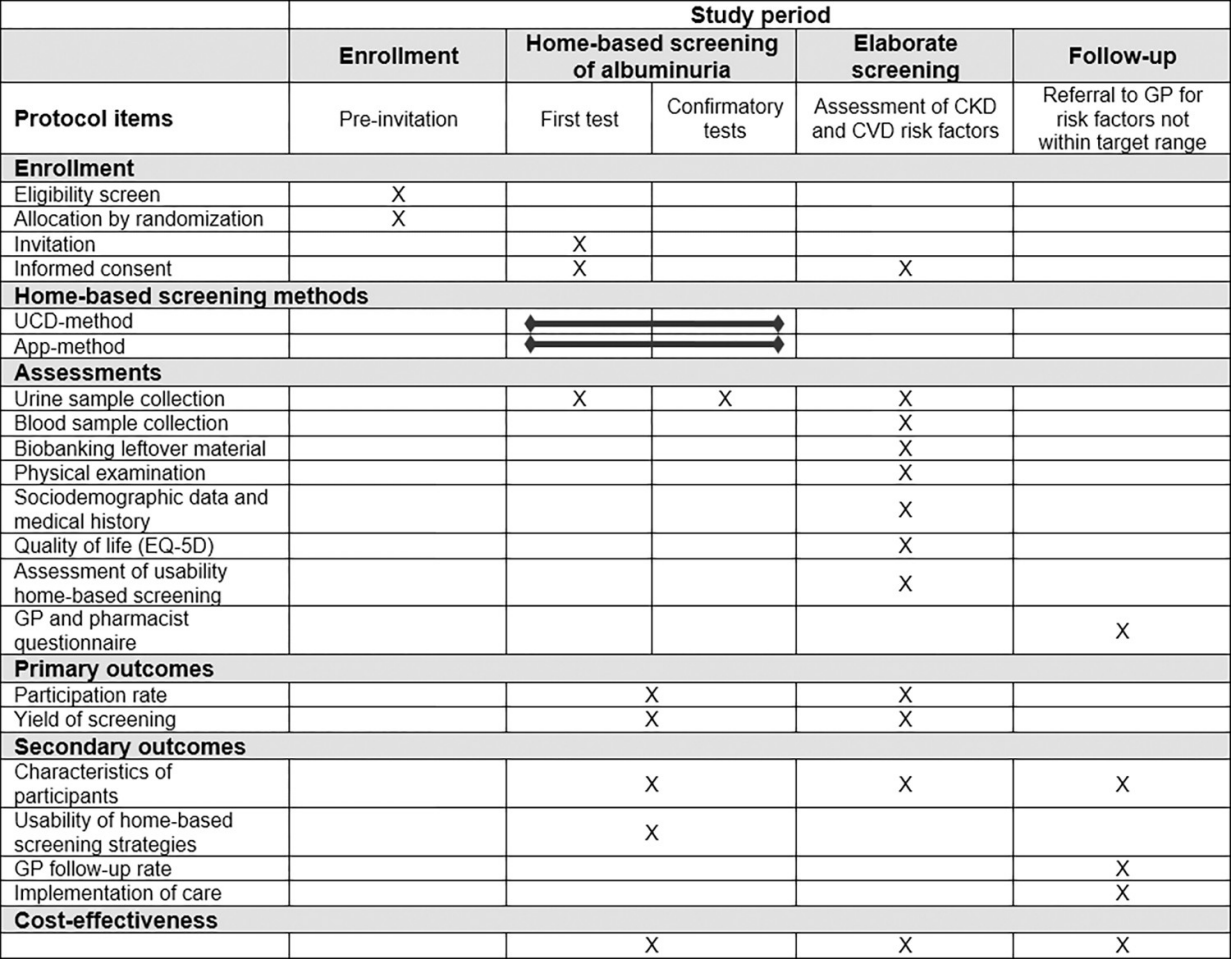
SPIRIT schedule of study procedures. Of note: Urinary sample collection includes the assessment albumin, creatinine and the ACR. Blood sample collection includes the assessment of HbA1c, glucose, total cholesterol, HDL cholesterol, LDL cholesterol, triglycerides, creatinine, and eGFR by CKD-EPI. Abbreviations: ACR, albumin-to-creatinine ratio; HDL, high-density lipoprotein; LDL; low-density lipoprotein; CKD-EPI, Chronic Kidney Disease Epidemiology Collaboration; CKD, chronic kidney disease; CVD, cardiovascular disease; GP, general practitioner; UCD, urinary collection device; App, smartphone application; EQ-5D, EuroQol 5D-5L.

**Fig 2:**
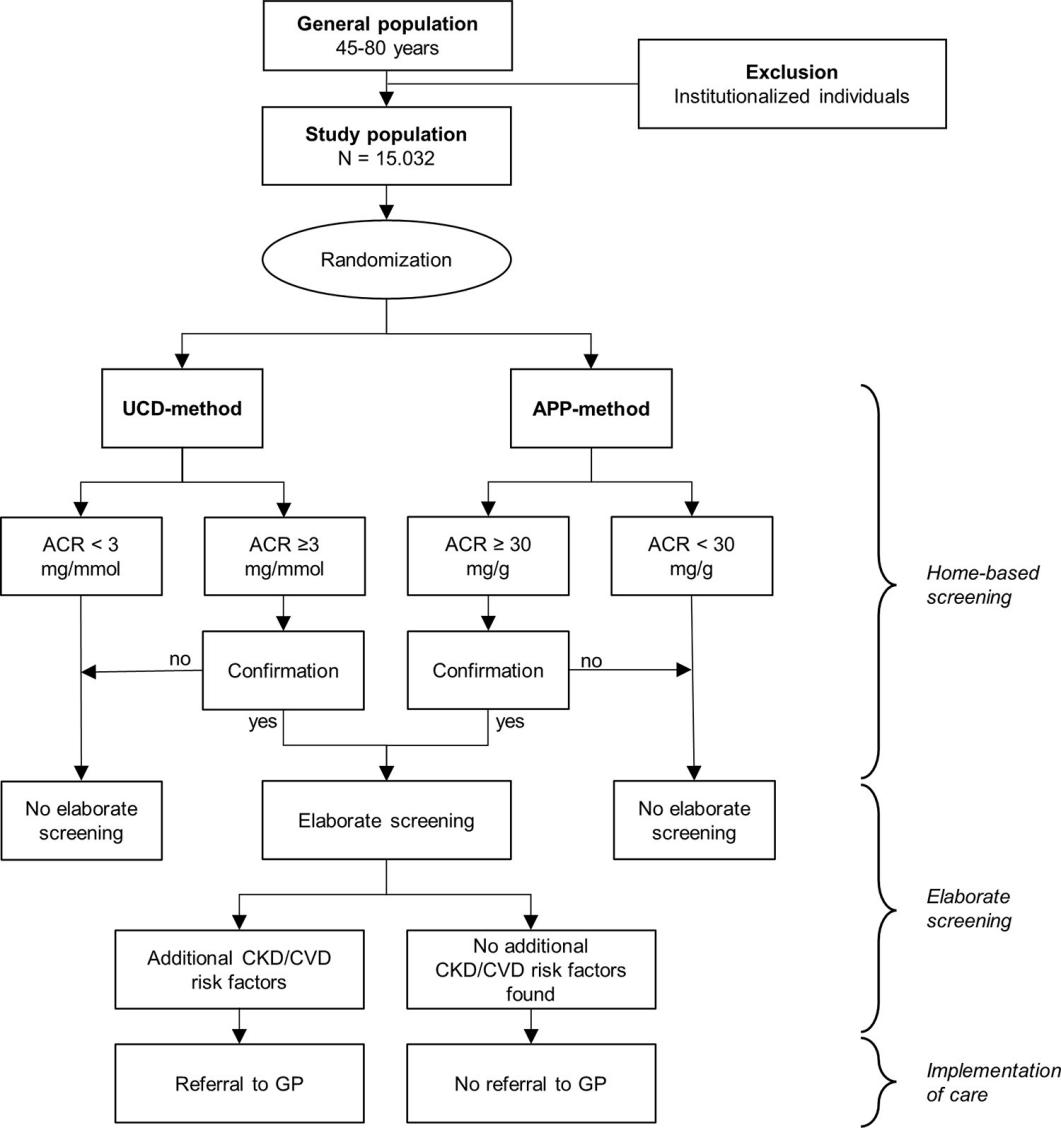
Study flow chart. Abbreviations: UCD, urinary collection device; App, smartphone application; ACR, albumin-to-creatinine ratio; CKD, chronic kidney disease; CVD, cardiovascular disease; GP, general practitioner.

An overview of the study design is shown in Figs 1 and 2. Individuals are randomized in a 1:1 ratio to be invited for home-based screening for albuminuria with either a more conventional urine collection device (UCD) or a smartphone application (App). Randomization is performed by an independent clinical research organization that is unaware of the characteristics of the individuals that are assigned one of the two methods for home-based screening for albuminuria. A 4-block randomization method is used, with randomization performed in batches of 5,000 individuals. Individuals living in one household are randomized to the same group. A unique participant identification code is assigned to all participants. Due to the nature of the study and used study materials, blinding of the participants for the assigned screening method is not feasible.

Before the start of the study, GPs and nurse practitioners (NPs) in general practice working in the screening region were informed about the study. During the study, a regional GP specialized in CKD and CVD serves as contact person for GPs and NPs. A website (www.niercheck.nl), telephone number, and e-mail address are available for participants and healthcare professionals to provide information about the study and to answer questions. In order to increase reach among the population, study materials were tested by a panel of volunteers from a Dutch organization that assists in improving the understandability of official texts for subjects of low literacy (The ABC Foundation).

#### Home-based screening

**Fig 3:**
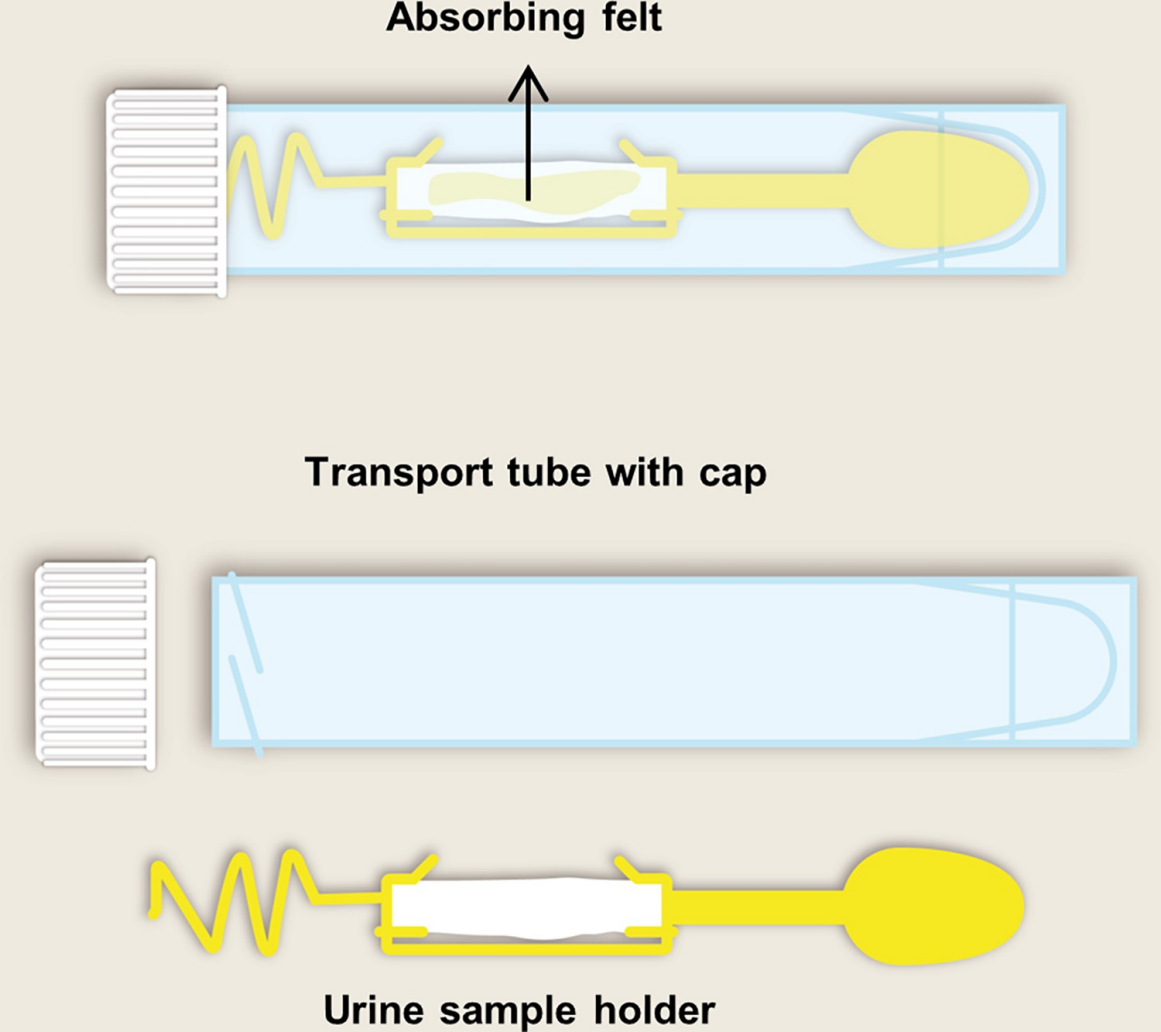
Urine collection device and its components to be used in the UCD-method. Abbreviations: UCD, urinary collection device.

All randomized individuals receive an invitation to participate in the home-based screening via mail, along with information on possible advantages and disadvantages of participation, and the assigned home-based screening method including test instructions. Participants are instructed to use midstream early morning void urine (EMV) for the test, and to postpone the home-based urine test in case of fever, urinary tract infection, pregnancy, menstruation, or having exercised intensively the day before. Two home-based albuminuria screening strategies are examined:

*UCD-method*. Individuals randomized to the UCD-method receive a PeeSpot UCD (Hessels+Grob B.V., Deventer, The Netherlands) [17,18]. This device consists of a holder containing a urine absorption pad in a transport tube (Fig 3). The urine absorption pad is a felt containing a dried hygroscopic polymer. Subjects hold the absorbent pad in their urine stream that absorbs approximately 1.2 ml of urine to collect a portion of urine. The participants insert the holder with the pad containing urine back into the tube, place the tube in a safety bag, and send this safety bag to a central laboratory by surface mail together with a signed informed consent form. A dried preservative in the urine absorption felt prevents bacterial growth during four days at room temperature. Participants are instructed to collect and send their urine sample on the same day, Monday through Thursday, and only during weather conditions that allow sending biosamples by mail (temperature between 0°C and 25°C). After receiving the sample in the central laboratory, the tube is centrifuged (5 minutes, 1800G) to release the urine into the tube with 100% albumin recovery. Urinary albumin and creatinine concentrations are measured by immuno-turbidimetry and enzymatically, respectively, on a cobas c502 analyzer (Roche Diagnostics, Almere, The Netherlands), with for albumin a lower limit of detection of 3 mg/L and intermediate precision of 4.2%, and for creatinine a lower limit of detection of 100 μmol/L and intermediate precision of 2.1%. ACR is reported as mg/mmol.

**Fig 4:**
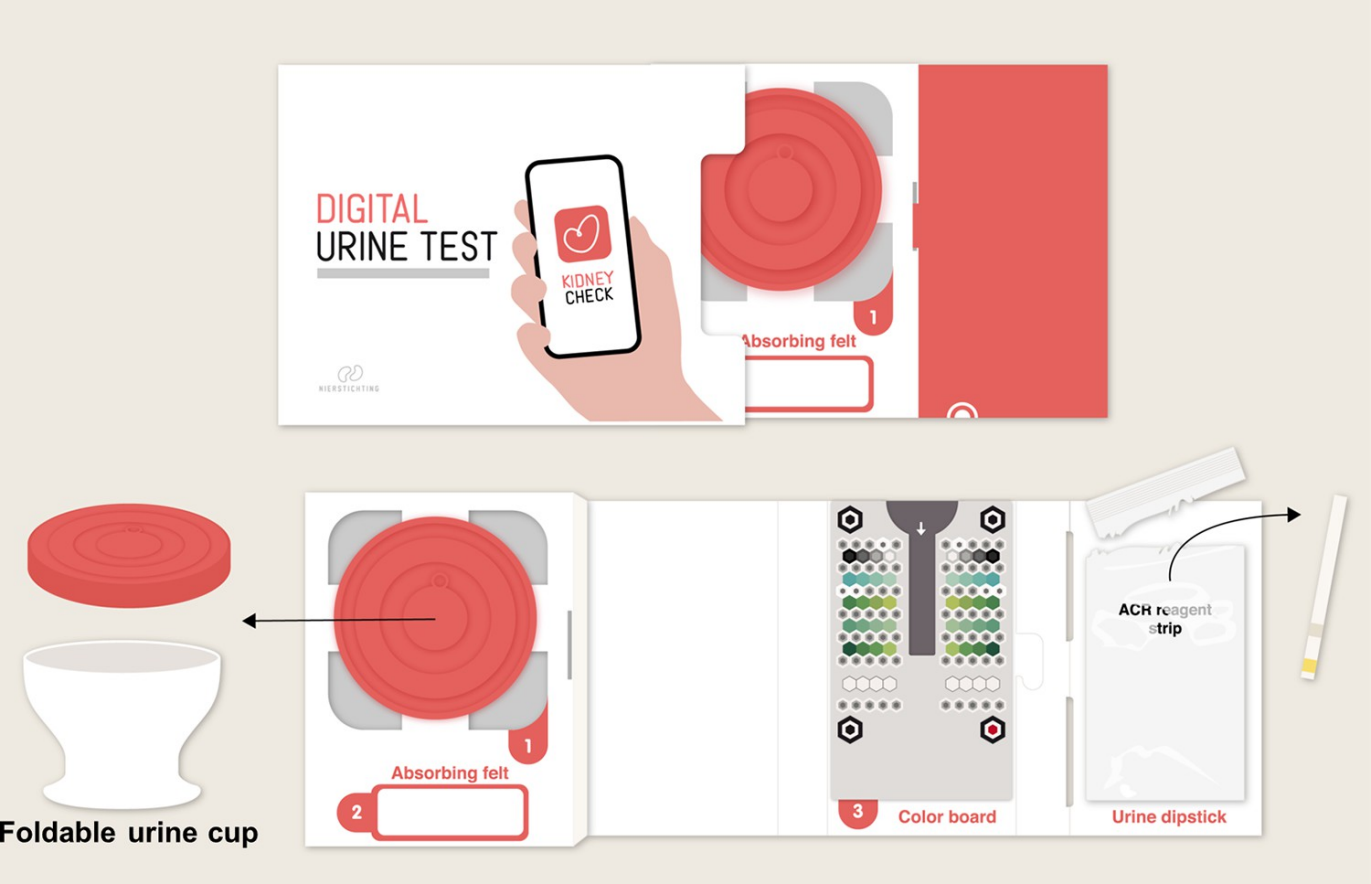
App test kit and its components to be used in the App-method. Abbreviations: App, smartphone application.

*App-method*. Individuals randomized to the App-method receive the ACR | EU test kit, a CE-marked home-based albuminuria screening self-test (Healthy.io Ltd, Tel Aviv-Yafo, Israel). This test kit contains a foldable urine cup, an ACR dipstick, an absorbing pad, a color board, and instructions to download a smartphone application (Fig 4). After providing digital informed consent, the App guides the user through the testing process step-by-step. Subjects first collect urine in the cup, in which they immerse the urine dipstick, of which the chemical pads change in color in response to the presence of albumin and creatinine. After the dipstick is placed on the color board, participants scan the dipstick with the App, which automatically makes a photo using the flashlight. The image is analyzed by an algorithm that uses the color board to calibrate for light conditions and color settings between different smartphone models. The App measures albumin at a concentration ≥ 6 mg/L as well as creatinine ≥ 0.06 g/L and returns results for the ACR following the KDIGO categorization: A1 (<30 mg/g, normal), A2 (30–300 mg/g, moderately increased), A3 (>300 mg/g, severely increased). This test result is directly shown to the participant in the App. Sensitivity and specificity for the home-based ACR | EU test are 98.4% and 92.9%, respectively, in a method comparison to the ACON U120 Ultra urine reagent strip analyzer (ACON Laboratories, San Diego, United States of America) that is routinely used in clinical chemistry labs.

If the first test result is negative (ACR < 3 mg/mmol for the UCD-method; ACR <30 mg/g for the App-method), no further examinations are needed. However, if the first test result indicates increased albuminuria, participants are sent a second test kit for confirmation. When the result of the second test is negative, a third test is sent. In case the initial increased albuminuria result is confirmed by either the second or third test, the participant is invited for a visit to a screening facility where an elaborate screening follows. Reminders are sent to non-participating subjects three and six weeks after the initial invitations.

#### Elaborate screening

Participants are asked not to perform any strenuous physical activity on the day before the screening and to complete a questionnaire. During the elaborate screening visit, height, weight, and blood pressure are measured, blood is drawn, and a spot urine sample collected. Blood pressure (mm Hg) and heart rate are measured four times using an automatic device (OMRON M7; OMRON Healthcare, Hoofddorp, The Netherlands) in a seated position on the non-dominant arm, of which the average of the last three measurements is reported. HbA1c, glucose, lipid profile, and creatinine are assessed in the blood samples. In the urine sample, albumin and creatinine are measured, and the ACR is calculated. Leftover urine and blood are stored in a biobank. The time of urine and blood sampling as well as fasting state of the participant are reported. During the elaborate screening, an internist-nephrologist is available for urgent medical questions. An overview of the measurements performed at the elaborate screening is provided in Fig 1.

Risk factors are defined as abnormal in accordance with prevailing guidelines for Dutch GPs. Overweight is defined as a body mass index (BMI) >25 in case of age ≤70 years, and a BMI >28 in case of age >70 years. Hypertension is defined as a systolic blood pressure ≥140 mm Hg, diastolic blood pressure ≥90 mm Hg, and in case of a positive ACR, a systolic blood pressure ≥130 mm Hg and diastolic blood pressure ≥80 mm Hg [19]. Type 2 diabetes is defined as fasting plasma glucose ≥7.0 mmol/L, non-fasting plasma glucose ≥11.1 mmol/L, or a HbA1c ≥48 mmol/mol, (also in accordance with the American Diabetes Association (ADA) Guideline for diabetes [20]). For patients with known type 2 diabetes, inadequate regulation is defined as an HbA1c >53 mmol/mol in case of age <70 years, >58 mmol/mol in case of age >70 years and duration diabetes <10 years, and >64 mmol/mol in case of age >70 years and duration diabetes >10 years [19]. Prediabetes is defined as fasting plasma glucose of 5.6–6.9 mmol/L or HbA1c of 39–48 mmol/L in accordance with the ADA Guideline for diabetes [20]. An unfavorable lipid profile is defined as low-density lipoprotein (LDL) cholesterol >3.0 mmol/L in case of no other positive risk factors, >2.6 mmol/L in case of positive ACR or diabetes, or >1.8 mmol/L in case of cardiovascular disease history and age ≤70 years [21]. Impaired kidney function is defined as an eGFR (using the creatinine-based Chronic Kidney Disease Epidemiology (CKD-EPI) Collaboration equation) <60 ml/min/1.73 m^2^ [22,23].

Participants and their GP receive the results of the elaborate screening by mail. When abnormalities are found that warrant treatment according to the prevailing GP guidelines, participants are referred to their GP to be prescribed lifestyle measures (e.g., to lose weight and/or stop smoking) and/or medical intervention. This is also the case when participants are already known with having these risk factors, but appear to be poorly controlled. If no abnormalities are found other than albuminuria, participants receive a letter with the screening results and a recommendation to visit their GP one year later for repeat screening of albuminuria and CVD risk factors, again in accordance with prevailing guidelines.

Participants invited to the elaborate screening also receive a questionnaire to collect data on demographics (including educational and ethnic background), medical history (presence/history of CKD, hypertension, diabetes, hypercholesterolemia, and CVD), and medication use. Other topics addressed by this questionnaire are quality of life (EuroQoL 5D-5L [24,25]), usability of the screening strategy, health literacy (the All Aspects of Health Literacy Scale (AAHLS) [26]), and contact details of their GP and pharmacy.

#### Follow-up

After the screening period, the GPs and pharmacists of participants who receive the advice to contact their GP for prescription of lifestyle advice or medication will be approached to confirm whether the participant visited their GP within six months after the elaborate screening. If so, the GP/pharmacist is asked whether this visit to the GP had led to the start or change of treatment (lifestyle and/or medication) of CKD and CVD risk factors. Information from GPs and pharmacists will be collected via a questionnaire. All GPs and pharmacists of participants of the elaborate screening who provided informed consent for contacting the GP and pharmacy are approached.

#### Informed consent procedures

All participants have to provide informed consent upon participating in the screening. For the UCD-method, participants receive an informed consent form in the same postal package as their screening test, which they must fill out and send back via a return envelope. For the App-method, participants have to complete an informed consent form via the App they have downloaded. This procedure is executed with the help of a signing service provider. For authentication, participants must first fill in their name and e-mail address in the App. Subsequently, they have to agree with the conditions stated in the informed consent by clicking on an ‘I agree’ button. Subsequently, a PDF-file containing an electronic signature and details is sent via e-mail to the participants and a copy to the research team. After this procedure, participants can start the home-based screening test. For the elaborate screening, additional informed consent is requested for contacting the participants’ GP and pharmacy and for storing leftover blood and urine samples for future analyses.

### Outcomes

#### Primary outcomes

The primary outcomes are the participation rate and yield of the home-based screening, the elaborate screening, and the overall screening program (Fig 1). The outcomes will be assessed separately for each of the two home-based screening strategies. The participation rate is defined as the number of individuals completing the home-based screening, elaborate screening, and overall screening program relative to the number of invited individuals (with completion including the required confirmation tests). The yield of the home-based screening is defined as the number of individuals who test positive for albuminuria (at least two tests positive) relative to the number of participating individuals. The yield of the elaborate screening is determined as the number of participants with increased albuminuria and newly diagnosed or poorly controlled CVD or CKD risk factors (including hypertension, hypercholesterolemia, type 2 diabetes, and impaired kidney function.

#### Secondary outcomes

As secondary outcome, the characteristics of participants and non-participants will be compared. Furthermore, the usability of the home-based screening strategies will be assessed based on user preferences, satisfaction rank, process evaluation, and usability success (measured as the percentage of people that tried to do a test and succeeded). Additionally, the GP follow-up rate will be assessed, defined as the number of participants visiting their GP after referral, relative to the number of referred individuals. In an exploratory analysis, the implementation of care by GPs will be assessed, defined as start or change of treatment (lifestyle advice or pharmacological treatment) in individuals visiting their GP after referral.

#### Cost-effectiveness

The cost-effectiveness of the screening is defined as the incremental cost-effectiveness ratio (ICER) in euros per Quality Adjusted Life Years (QALY) gained for each of the two screening strategies compared to the standard of care. The ICER will be modeled using data obtained in the study and treatment efficacy assumed from data in literature.

#### Sub-studies

Two sub-studies will be performed. The first sub-study will examine the false-negative rate for both home-based albuminuria screening strategies. Around 500 subjects per screening method who have a negative first home-based albuminuria screening test will be invited to perform an additional test, to examine the false-negative rate. This additional test will be done via the UCD, as this test provides a quantitative, exact ACR value as usual in clinical care. There is no need for further action, if this second test is also negative. If this second test is positive, the participant is sent a third test to confirm the result.

The second sub-study will examine whether lowering the cut-off value for the ACR increases the yield of the home-based screening. All subjects of the UCD-method with an ACR in their first test between 2–3 mg/mmol are invited for a second home-based albuminuria test (again with the UCD). If the ACR of the second test is negative (ACR <30 mg/g), there is no need for further action. If the ACR of the second test is positive (ACR ≥30 mg/g), the participant is invited for a third home-based test. If the third test is negative, no further action is undertaken. If the ACR of the third test is positive, the participant is invited for the elaborate screening.

### Data management

A secured data platform is designed that contains unique participant IDs, matched with the names and addresses of the participants. A second, secured data platform is designed to enable pseudonymized data transfer and data storage. In this data platform, a computerized record is kept of all home-based screening test results to enable correct dispatching of test results and reminders, and to send confirmation screening tests and invitations for the elaborate screening visit if appropriate. Questionnaires filled out by the participants are sent to the University Medical Center Groningen research team and are recorded digitally in an electronic case report form (eCRF) in a certified Electronic Data Capture data management infrastructure (REDCap Research Electronic Data Capture, version 10.0.23, Vanderbilt University). Trained investigators perform the data entry.

### Sample size

The sample size of this study is calculated to be able to show a 5% difference in participation rate between the two screening strategies, with assumptions based on previous data [7], including an overall participation rate for the home-based screening of 50%, 8% of the participants having elevated ACR, of which 90% will be confirmed by the confirmatory tests, and a participation rate in the elaborate screening of 90%. Adopting a power of 80% and a 2-sided alpha of 0.05, a minimum of 15.032 individuals are to be invited to participate to detect this 5% difference in participation rate between the two screening strategies. This number of individuals also allows discerning a significant difference between both screening strategies in the number of individuals with elevated albuminuria diagnosed with at least one newly discovered CKD or CVD risk factor.

### Statistical analyses

The statistical package IBM SPSS (version 23, Armonk, NY) will be used for the analysis of the data. Baseline characteristics for continuous variables will be expressed as mean and standard deviation for continuous variables in case of normal distribution, and as median and interquartile range in case of non-normal distribution. Discrete variables will be reported as number and proportions. The primary outcomes, participation rate and yield of screening, will be compared between the two treatment strategies using the Chi-square test. Participation and completion rates will be presented for the intention-to-screen population. Yield of screening will be presented for the intention-to-screen population as well as the per-protocol population. Characteristics of participants and non-responders will be compared using univariable and multivariable logistic regression models. Cost-effectiveness will be assessed using an individual-level health state transition model (microsimulation), comparing the two screening strategies with usual care (no screening). It will be carried out from a Dutch healthcare perspective and the model will be run over the remaining lifetime. Data from literature will be used to estimate participants quality of life, costs of interventions after the elaborate screening, the efficacy of interventions to prevent CVD and CKD progression, and the costs associated with CVD and CKD events. Effectiveness will be calculated expressed in total costs and total effects (in QALYs) per screening method. Incremental effects will be calculated and the incremental cost-effectiveness ratio (ICER), will be determined, defined as incremental costs divided by incremental gain in QALYs.

### Ethics approval

Institutional review board approval of the study protocol was obtained by the Medical Ethics Committee of the University Medical Center Groningen, Groningen, the Netherlands (2018.687). The study is designed and performed in accordance with the International Conference of Harmonization Good Clinical Practice Guidelines and with the principles of the Declaration of Helsinki.

### Study organization and study status

An advisory board including healthcare professionals from primary and secondary care will be consulted for advice on the design and conduct of the study. A central study team from the University Medical Center Groningen coordinates the study, with support from a regional team for local logistical issues. An independent and qualified data monitor will verify the participants’ rights, well-being, and safety, as well as the quality of the data.

The study started in November 2019. A total study time of one year for the home-based screening and elaborate screening was foreseen, but due to the COVID-19 pandemic, the study was temporarily halted and therefore delayed. Data collection is ongoing at the moment.

## Discussion

The present study will evaluate the participation rate, yield and cost-effectiveness of home-based screening for albuminuria in the general population. The importance of screening for albuminuria has been emphasized in recent years, as elevated albuminuria has increasingly been recognized as an early marker for both progression of CKD and generalized vascular endothelial damage. This holds, however, for patients with known type 2 diabetes or hypertension. Currently, only low-quality evidence is available on the benefits and harms of population screening for albuminuria in asymptomatic individuals. The THOMAS study is designed to address this knowledge gap prospectively.

In this screening study’s design, several choices were made that merit discussion. Regarding the study region, the region of Breda was chosen because the characteristics of its population closely resemble those of the average Dutch population concerning sex, age, and socioeconomic status distribution [16]. Its settings also reflect most Dutch towns, with an urban center harboring around 200.000 inhabitants and rural surroundings. Moreover, this region has not yet been subjected to large-scale screening efforts in the past.

Regarding the age thresholds of the study population, limiting screening for albuminuria to those aged ≥50 years has been suggested to be the most cost-effective considering the age-dependent prevalence of CKD [13,14]. As advancing age might reduce the chance that the benefits of screening will exceed its harms, most of the Dutch population screening programs use the age of 75 years as upper age limit, for instance for colorectal as well as for breast cancer screening [27,28]. In the present study, a slightly wider age range of 45–80 years was chosen to investigate the most cost effective and cost-effective age range.

It was decided to invite the total general population for the screening. It could be considered to screen only subjects not known yet with CKD and CVD risk factors. Indeed, in such patients, the benefits of screening may be most optimal. However, logistically it is difficult to delineate which subjects should be invited in this scenario. In the Netherlands, as in most countries, information on CKD and CVD risk profile is not readily available in a central database because of privacy considerations. Additionally subjects with known, but poorly controlled risk factors will be found that will benefit from optimizing treatment, by inviting the general population. Furthermore, by inviting the general population, the study will provide up-to-date information concerning albuminuria and CKD prevalence in the Netherlands.

As discussed earlier, the cost-effectiveness of albuminuria screening performed by GPs has been questioned [12–14]. Therefore, we designed a home-based albuminuria screening strategy with subsequent screening by GPs of only those with proven elevated albuminuria. With such a strategy, the burden on primary care will be reduced. Moreover, the costs will be lower, likely resulting in a cost-effective scenario [15].

In the present study, two home-based methods to assess albuminuria will be investigated. The UCD-method has the advantage of accurate measurement of albuminuria with immuno-turbidimetry, whereas the App-based dipstick method has the advantage of being able to communicate directly with participants on the results and consequences of these results, which may optimize participation. Both methods are easily accessible strategies for investigating albuminuria in the home situation.

It was decided to use an EMV sample and not 24-hour urine collections for the measurement of albuminuria. Although assessment of 24hr urinary albumin excretion is considered the gold standard for detecting albuminuria, collecting a 24-hour urine sample is not feasible for large scale population screening because it is time-consuming and inconvenient for participants. Instead, an EMV sample was chosen to measure the ACR, as this is more acceptable and shown to be better correlated with 24hr UAE than ACR in a random spot urine sample [29].

The ACR cut-off to indicate elevated albuminuria was set at 30 mg/g and 3 mg/mmol, in accordance with the prevailing KDIGO guideline [23]. The present study will, however, also investigate in a sensitivity analysis the effect of lowering the cut-off value for the first ACR test on the yield of the home-based screening. Such a strategy may lower the number of false negatives but on the other hand, increase the false positivity rate.

For the determination of the sample size, an overall participation rate of 50% is assumed. This is based on the experience from the PREVEND study. This observational, epidemiological study started in 1997 and aimed to study the natural course and consequences of elevated albuminuria in the general population. In total, 48% of the invited population participated and sent a test tube containing some urine collected at home to a central laboratory [7].

There are other important considerations that merit attention. First, this screening study is primarily focused on early identification of subjects with CKD. Therefore, not all subjects with CVD risk factors will be identified. Previous epidemiological analyses showed that people with newly discovered CVD risk factors, but no elevated albuminuria, on average have an absolute risk for CVD that does not merit start of cardioprotective treatment [11]. In contrast, subjects with newly discovered CVD risk factors and elevated albuminuria in general have such a high CVD risk that start of preventive treatment is indicated [11]. These data suggest that a screening primarily directed at discovering subjects with elevated albuminuria may be effective in discerning high-risk subjects for whom screening will have therapeutic consequences. Future studies could further investigate this. Second, the present study investigates the effectiveness of a single screening. Therefore, this study cannot directly demonstrate potential benefits regarding renal and CV event outcomes for repeated screening. However, the effectiveness of a repeat screening strategy can be modeled in the cost-effectiveness analyses. Third, it is assumed that the potential harms of the study are limited, because our screening strategy does not include invasive procedures. In addition, the psychological burden should be limited, because the diagnosis of CKD is based on an initial positive test that has to be confirmed, which by definition implies CKD. To further reduce potential psychological distress induced by the screening, all subjects will receive a brochure with detailed information including potential benefits and harms of participation. More information can be found on a dedicated internet site (www.niercheck.nl). Lastly, the UCD screening method uses the ACR cut-off of 3 mg/mmol for elevated albuminuria, whereas the App uses an ACR cut-off of 30 mg/g, which are not exactly equal. If needed, after completion of the study the results of the UCD-method can be modeled as if they were also reported in mg/g to evaluate any consequences and to allow a fair comparison with the App-method.

Several strengths regarding the study design can be mentioned. This is the first large-scale implementation study to prospectively investigate the participation rate, yield and cost-effectiveness of population-based screening for albuminuria for the early detection of CKD and CVD risk factors. The study uses a randomized approach, randomizing participants into one of the two screening strategies. Therefore, we can evaluate and compare both methods with respect to user-friendliness, willingness to participate, yield, and cost-effectiveness. This will allow an evidence-based choice for the most promising method for further exploration. The study design is further strengthened by the fact that albuminuria is not assessed only once in the home setting, but that confirmation tests are incorporated to reliably test whether albuminuria is elevated before participants are invited for a costly and more burdensome visit to a screening center for elaborate screening of CKD and CVD risk factors. This is in accordance with prevailing guidelines and takes variation in albuminuria into account, limiting the chance of false-positive findings [23].

To summarize, the THOMAS study is a unique investigator-initiated, population-based randomized trial that prospectively investigates the participation rate, yield and cost-effectiveness of screening for albuminuria for the early detection of CKD and CVD risk factors in the general population. By evaluating and comparing two home-based tests to assess albuminuria, insight will be provided to decide which strategy is the most promising to be explored further. Thus, the results of the THOMAS study will contribute to an improved, structured approach to the early detection of CKD and CVD risk factors.

## Supporting information

S1 File SPIRIT 2013 checklist. (DOC)

S2 File Study protocol approved by the medical ethics committee. (PDF)
