# Supplementary material for: Protocol for a randomized study assessing the feasibility of home-based albuminuria screening among the general population: The THOMAS study
Source: PLoS One. 2022 Dec 22;17(12):e0279321. doi: 10.1371/journal.pone.0279321 (PMC9778938; doi:10.1371/journal.pone.0279321)
Supplement: S2 File — (PDF) [file pone.0279321.s002.pdf]

THOMAS

‘Towards HOMe-based Albuminuria Screening: an implementation study testing two approaches’

Written by:
L.M. Kieneker, PhD
Prof. dr. R.T. Gansevoort
University Medical Center Groningen
Department Internal Medicine
Division of Nephrology (AA53)
Hanzeplein 1
P.O. Box 30.001
9700 RB Groningen
The Netherlands

Subsidiary party: Topsector Life Sciences & Health, Dutch Kidney Foundation

PROTOCOL TITLE

THOMAS: 'Towards HOMe-based Albuminuria Screening: an implementation study testing two approaches'

**Protocol ID**	NL65228.042.18
**Short title**	Towards home-based albuminuria screening
**Version**	5.3
**Date**	26-05-2020
**Principal investigator**	Prof. dr. R.T. Gansevoort<br>University Medical Center Groningen<br>Department of Internal Medicine<br>Division of Nephrology (AA53)<br>P.O. Box 30.001<br>9700 RB Groningen<br>The Netherlands<br>Telephone: 050-3619023<br>E-mail: r.t.gansevoort@umcg.nl
**Sponsor**	University Medical Center Groningen<br>P.O. Box 30.001<br>9700 RB Groningen<br>The Netherlands
**Subsidising party**	Health~Holland (Top Sector Life Sciences & Health) and the Dutch Kidney Foundation
**Independent expert**	Dr. C.F.M. Franssen, internist-nephrologist<br>University Medical Center Groningen<br>Department of Internal Medicine<br>Division of Nephrology (AA53)<br>P.O. Box 30.001<br>9700 RB Groningen<br>The Netherlands

**Laboratory sites**	Laboratory for Clinical Chemistry and Hematology<br>Amphia Hospital<br>P.O. Box 90158<br>4800 RK Breda<br>The Netherlands

PROTOCOL SIGNATURE SHEET

Name	Signature	Date
**Head of Department:**<br>Prof. dr. R.O.B. Gans, internist<br>Dept. Internal Medicine<br>UMC Groningen		
**Principal Investigator:**<br>Prof. dr. R.T. Gansevoort, nephrologist<br>Dept. Internal Medicine, section Nephrology<br>UMC Groningen		
**Study coordinator/investigator:**<br>Drs. L.M. Kieneker, PhD.<br>Dept. Internal Medicine, section Nephrology<br>UMC Groningen		

TABLE OF CONTENTS

1. INTRODUCTION AND RATIONALE 12
2. OBJECTIVES 14
3. STUDY DESIGN 15
4. STUDY POPULATION 20
   - 4.1 Population (base) 20
   - 4.2 Inclusion criteria 22
   - 4.3 Exclusion criteria 22
   - 4.4 Sample size calculation 22
5. INVESTIGATIONAL PRODUCT 24
   - 5.1 Name and description of investigational product 24
   - 5.2 Summary of findings from non-clinical studies 25
   - 5.3 Summary of findings from clinical studies 25
   - 5.4 Summary of known and potential risks and benefits 25
6. NON-INVESTIGATIONAL PRODUCT 26
   - 6.1 Name and description of non-investigational product 26
   - 6.2 Summary of findings from non-clinical studies 26
   - 6.3 Summary of findings from clinical studies 26
   - 6.4 Summary of known and potential risks and benefits 26
   - 6.5 Description and justification of route of administration and dosage 27
   - 6.6 Dosages, dosage modifications and method of administration 27
   - 6.7 Preparation and labelling of Non Investigational Medicinal Product 27
   - 6.8 Drug accountability 27
7. METHODS 28
   - 7.1 Study parameters 28
     - 7.1.1 Main study parameters 28
     - 7.1.2 Secondary study parameters 28
     - 7.1.3 Other study parameters 30
   - 7.2 Randomisation, blinding and treatment allocation 30
   - 7.3 Study procedures 30
   - 7.4 Withdrawal of individual subjects 34
   - 7.5 Replacement of individual subjects after withdrawal 34
   - 7.6 Follow-up of withdrawn subjects 34
   - 7.7 Premature termination of the study 34
8. SAFETY REPORTING 35
   - 8.1 Temporary halt for reasons of subject safety 35
   - 8.2 AEs, SAEs, and SADEs 35
     - 8.2.1 Adverse events (AEs) 35
     - 8.2.2 Serious adverse events (SAEs) 35
     - 8.2.3 Serious adverse device events (SADEs) 36
   - 8.3 Follow-up of adverse events 36
9. STATISTICAL ANALYSIS 37

9.1 Primary study parameter(s) 37
9.2 Secondary study parameter(s) 37
9.3 Other study parameters 39
9.4 Interim analysis 40
10. ETHICAL CONSIDERATIONS 41
10.1 Regulation statement 41
10.2 Recruitment and consent 41
10.3 Benefits and risks assessment, group relatedness 42
10.4 Compensation for injury 42
11. ADMINISTRATIVE ASPECTS, MONITORING AND PUBLICATION 43
11.1 Handling and storage of data and documents 43
11.2 Monitoring and Quality Assurance 43
11.3 Amendments 44
11.4 Annual progress report 44
11.5 Temporary halt and (prematurely) end of study report 44
11.6 Public disclosure and publication policy 44
12. STRUCTURED RISK ANALYSIS 45
12.1 Potential issues of concern 45
13. ADVISORY BOARD 46

LIST OF ABBREVIATIONS AND RELEVANT DEFINITIONS

**AAHLS**	**All Aspects of Health Literacy Scale**
**ABR**	**ABR form, General Assessment and Registration form, is the application form that is required for submission to the accredited Ethics Committee (In Dutch, ABR = Algemene Beoordeling en Registratie)**
**ACR**	**Albumin-to-creatinine ratio**
**AE**	**Adverse Event**
**AR**	**Adverse Reaction**
**CCMO**	**Central Committee on Research Involving Human Subjects; in Dutch: Centrale Commissie Mensgebonden Onderzoek**
**CKD**	**Chronic Kidney Disease**
**CV**	**Curriculum Vitae**
**CVD**	**Cardiovascular Disease**
**eCRF**	**Electronic Case Report Form**
**eGFR**	**Estimated glomerular filtration rate**
**EU**	**European Union**
**EQ-5D**	**EuroQol 5D-5L, a standardized questionnaire that provides a score at five health levels whereby a weighted health index can be derived for an individual**
**GCP**	**Good Clinical Practice**
**GP**	**General Practitioner**
**HDL**	**High-density lipoprotein**
**IC**	**Informed Consent**
**ICER**	**Incremental cost-effectiveness ratio**
**LDL**	**Low-density lipoprotein**
**METC**	**Medical research ethics committee (MREC); in Dutch: medisch ethische toetsing commissie (METC)**
**NFU**	**Netherlands Federation of University Medical Centres**
**PREVEND**	**Prevention of Renal and Vascular End-stage Disease**
**QALY**	**Quality-Adjusted Life Year**
**SAE**	**Serious Adverse Event**
**SADE**	**Serious Adverse Device Events**
**Sponsor**	**The sponsor is the party that commissions the organisation or performance of the research, for example a pharmaceutical**

	**company, academic hospital, scientific organisation or investigator. A party that provides funding for a study but does not commission it is not regarded as the sponsor, but referred to as a subsidising party.**
**UMC**	**University Medical Center**
**Wbp**	**Personal Data Protection Act (in Dutch: Wet Bescherming Persoonsgevens)**
**WMO**	**Medical Research Involving Human Subjects Act (in Dutch: Wet Medisch-wetenschappelijk Onderzoek met Mensen**

SUMMARY

Rationale: Chronic kidney disease (CKD) is a worldwide major public health problem that is associated with an increased incidence of kidney failure and cardiovascular morbidity and mortality, which causes high costs for society. Symptoms of CKD are aspecific and usually start to appear only when kidney function drops to below 30%. At that time preventive measures will have only limited efficacy. Elevated albuminuria has increasingly been recognized as an early marker of kidney damage, and is also associated with high blood pressure, diabetes, and high cholesterol levels, which are all important risk factors for progressive CKD and cardiovascular disease (CVD). Importantly, it has been shown that in the Dutch population, the prevalence of yet undiagnosed risk factors is considerably higher than the prevalence of known risk factors. Population screening for albuminuria could therefore detect a considerable number of subjects with yet unknown risk factors for progressive CKD and CVD who can benefit of early intervention. However, it is yet unknown whether the Dutch population accepts population screening for albuminuria, which method will be most suitable, and whether screening is cost-effective.

Objective: The aim of the present study is to examine whether population screening for increased albuminuria among the Dutch population will contribute to the early detection of albuminuria and other yet undiagnosed risk factors for CVD and CKD. We will do this by evaluating the participation rate, the characteristics of responders and non-responders, and the yield of the screening, i.e., number of subjects detected with elevated albuminuria (defined as albumin-to-creatinine ratio [ACR] >30 mg/g) and newly diagnosed risk factors for progressive CKD and CVD. We will also evaluate the cost-effectiveness of albuminuria screening. Moreover, we will evaluate two different screening techniques for albuminuria screening and investigate the difference in participation rate, characteristics of the responders, yield of the screening, and cost-effectiveness.

Study design: The study will be an observational study.

Study population: In total, a random sample of 15.032 subjects aged 45-80 years from the Dutch general population living in the region of Breda will be invited to participate in the study. Subjects will be randomized 1:1 into approach A (albuminuria screening using the ACR performed in the central laboratory after urine collection with a PeeSpot urine collection device) or approach B (albuminuria screening using the ACR | EU self-test).

Design: Participants randomized to approach A will receive the PeeSpot urine collection device. This device consists of a holder containing a urine collection pad consisting of an absorption felt containing a dried hygroscopic polymer and a preservative. Urine collected in this device (midstream, early morning void) is sent to the laboratory (Amphia Hospital, Breda) for measurement of the ACR. Participants randomized to approach B will receive the ACR | EU self-test kit, which consists of a cup, a test strip, a color board, and instructions to download a smartphone application. For this test, subjects have to collect urine in the cup

(midstream, early morning void), immerse the test strip into urine, of which the chemical pads will change in color in response to the presence of albumin and creatinine. The stick is placed on the color board and scanned with the application, which uses flash light. The app automatically analyzes semi-quantitatively an ACR (normal, abnormal, high abnormal). If the test result (either PeeSpot or ACR | EU self-test) is positive (i.e., elevated albuminuria), participants will be sent an extra test of the same type as initial measurement for confirmation. When the test result of the second test is normal (indicating no elevated albuminuria), participants will receive a third test. When elevated albuminuria is confirmed by at least 1 of the confirmatory tests (in accordance with prevailing guidelines), the participant will be referred for further screening for CKD and CVD risk factors (hypertension, diabetes, hypercholesterolemia, impaired kidney function) at a central screening facility (Amphia Hospital, Breda). Depending on the results of the elaborate screening, subjects will be referred to their general practitioner to be prescribed lifestyle measures and/or medical intervention, again, in accordance with prevailing guidelines.

Main study parameters/endpoints: The main study parameters are participation rate, the yield of screening i.e., number of subjects detected with elevated albuminuria (ACR >30 mg/g) and newly diagnosed risk factors for progressive CKD and/or CVD (hypertension, diabetes, hypercholesterolemia, impaired kidney function), characteristics of responders and non-responders, and the usability (assessed by a questionnaire), and the cost-effectiveness albuminuria screening. We will also investigate whether there are differences in these parameters between the two screening methods (PeeSpot vs. ACR | EU).

Nature and extent of the burden and risks associated with participation, benefit and group relatedness: Both screening methods are non-invasive tests, that do not have associated physical risks. When elevated albuminuria is observed and confirmed, subjects are invited for further screening in a central screening facility (Amphia Hospital, Breda). During this visit, blood pressure measurements will be performed and 7 mL blood will be drawn for assessment of blood glucose, HbA1c, total, high-density lipoprotein (HDL), low density lipoprotein (LDL) cholesterol, triglycerides, and creatinine, and an additional spot urine sample will be collected for ACR measurement using standard lab technology. Leftover blood and urine will be stored coded at -80°C for possible further analyses in line with the present study.

In addition, a subset of the participants will, at the end of the study receive a questionnaire including questions on demographics, educational level, disease history, medication use, quality of life, and usability of the screening test. This subset of participants include: 1) all subjects with an overall positive test for albuminuria (at least 2 out of 2 or 3 ACR tests positive) invited to the elaborate screening and 2) a subset of subjects with an initial negative ACR test who will be asked to perform a second and third test to examine the false-negative rates (N=500 for both arms).

The risks associated with the study are low, but the burden may be 1) confrontation with an unfavorable result, 2) unnecessary anxiety in case of false-positive test results, and 3) unwarranted reassurance in case of false-negative results. Benefit consists of the possibility

that risk factors for progressive CKD and CVD are detected in an early phase, allowing timely start of preventive strategies.

1. INTRODUCTION AND RATIONALE

Chronic kidney disease (CKD) is a worldwide major public health problem that is associated with an increased incidence of kidney failure and cardiovascular events (1, 2). The economic and societal burden of CKD is high, because of the burden on the work force (loss of working capacity) and the high health care costs involved (3). CKD causes aspecific symptoms, such as fatigue and impaired physical functioning, that appear only when kidney function is decreased to less than 30%. At that time preventive measures will have only limited efficacy. To tackle this burden, screening for CKD among the general population has been proposed to allow early detection and treatment of CKD.

CKD can be assessed by either showing impaired kidney function (eGFR) or elevated albuminuria. Whereas in the past most attention was focused especially on impaired kidney function, more recently attention has shifted towards albuminuria. Reason for this change is that screening for impaired kidney will allow only late intervention (because subjects are already near the need for kidney function replacement therapy). In contrast, many subjects with elevated albuminuria still have normal kidney function, allowing early start of preventive treatment, as shown in Figure 1.

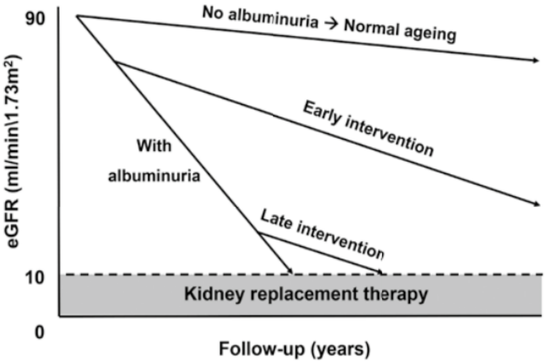

Figure 1. Schematic presentation of kidney function decline over the years and the effect of late versus early intervention to prevent the need for kidney replacement therapy. Adapted from Gansevoort and de Jong (2009).

In the last decades, elevated albuminuria has increasingly been recognized as a marker of generalized vascular endothelial damage. As such it is not only an early predictor of progressive CKD, but also of progressive cardiovascular disease (CVD) (4-6). In a previous study, the majority (65%) of subjects found by screening to have increased albuminuria also had hypertension, diabetes, or hypercholesterolemia. In two-thirds of these subjects, these abnormalities were not yet diagnosed (7). Early detection of increased albuminuria could be of help for prevention because it gives the opportunity to treat the risk factors for CKD and CVD in an early stage. Population screening for albuminuria has been proposed. Such a screening can be justified according to the WHO criteria of Wilson and Jungner (8), because CKD has important consequences for subjects, the initially symptomless course of the disease, and the availability of effective treatment. However, implementation research evaluating methods that have been proposed to screen the general population for risk factors for CKD and CVD is lacking, as are cost-effectiveness studies.

In 2006, an awareness campaign was organized by the Dutch Kidney Foundation named the Niercheck. Subjects from the general population could order a dipstick to measure the (macro)albuminuria concentration of their urine, as a measure of kidney damage. Anticipated

was that around 150.000 subjects would order such a dipstick, but it turned out that 1.6 million subjects did so, showing a great interest among the Dutch population. In total, 20% of the participants tested positive for albuminuria. However, there were many with a false positive test. This was due to the difficulty to accurately read the dipsticks caused by the different lighting conditions and noncompliance to performance instructions, i.e. time between dip and reed, and by the fact that subjects did not confirm an initial positive test before seeking help. Therefore, this method is not suited for population screening. Recently, two novel methods have been developed, that hold promise to be used for population screening as it tackles the aforementioned issues.

The first method has been developed by Healthy.io, an Israeli e-health company, and is named the ACR | EU test. This test is an albumin-to-creatinine ratio (ACR) dipstick in combination with a smartphone application that is able to accurately measure the ACR by standardizing the light source. The ACR | EU test builds on Dip.io test –Healthy.io's urinalysis product with 10 parameters including proteinuria– which already received significant support within the international Nephrology community (collaborations with the US National Kidney Foundation [Geisinger Health], and a general practitioner partnership in the UK). Healthy.io adapted its Dip.io into the ACR | EU test for the present study to allow screening for microalbuminuria (leakage of very small proteins in the urine) instead of proteinuria (leakage of bigger proteins in the urine). This novel method might be suited for population screening for albuminuria. The second method is the PeeSpot urine collection device with analysis of ACR in the central laboratory. This is a plastic tube containing a holder with a collection pad absorbing urine. People can collect a sample of urine with this device and this can be send to a central laboratory for measurement of the ACR by the standard method, i.e., albumin by immunotubidimetry and creatinine by specific enzymatic assay. A detailed description of both devices is provided in Chapters 5 and 6. Both devices have their advantages and disadvantages for screening. For instance, it might be that the smartphone application is better suited for young subjects with higher education, whereas the PeeSpot method is better accepted by elderly and people with low (health) literacy.

In the current study, we will investigate whether population screening for increased albuminuria among the Dutch population can contribute to the early detection of yet undiagnosed risk factors for CKD and CVD. We will evaluate both the ACR | EU self-test and the PeeSpot-central lab-method for the detection of increased albuminuria, by examining among others the participation rate, the characteristics of the responders and non-responders, evaluating the usability of the screening techniques, and assessing the yield of the screening (i.e., number of subjects detected with elevated albuminuria and newly diagnosed risk factors of CKD and/or CVD). Finally, we will evaluate the cost-effectiveness of albuminuria screening.

2. OBJECTIVES

Primary Objective:

To investigate whether population screening for increased albuminuria among the Dutch population can contribute to the early detection of yet undiagnosed risk factors for renal and cardiovascular diseases in an early stage. For this, we will investigate:

1. The participation rate of the screening;
2. The yield of albuminuria screening (number of subjects with elevated albuminuria [ACR >30 mg/g] and with newly diagnosed risk factors including hypertension, diabetes, hypercholesterolemia, and impaired kidney function), and;
3. To evaluate the cost-effectiveness of albuminuria screening and compare this to standard of care.

Secondary Objectives:

1. To assess differences in participation rate, yield, and cost-effectiveness between the two different screening methods (PeeSpot test vs. ACR | EU test).
2. To asses the characteristics of the participants, including differences in age, sex, educational level, estimated social economic status (based on data of Statistics Netherlands, providing estimated social economic status based on postal codes), medication use, and history and presence of disease (obtained by a questionnaire);
3. To asses the characteristics of the non-responders, including differences in age, sex, and estimated social economic status (the latter is based on data of Statistics Netherlands, providing estimated social economic status based on postal codes);
4. To examine differences in characteristics of the responders and non-responders of the two different screening methods (PeeSpot vs. ACR | EU).
5. To assess the usability of the two different screening methods (PeeSpot vs. ACR | EU).
6. To evaluate the best ACR cut-off value to achieve the highest yield of the screening.
7. To examine the difference in in rate of previously undiagnosed risk factors (hypertension, diabetes, hypercholesterolemia, and impaired kidney function) that were found at the elaborate screening between subjects who have albuminuria and subjects without albuminuria.

3. STUDY DESIGN

A schematic presentation of the study is shown in Figure 2. In total, 15.032 subjects aged 45-80 years from the Dutch general population living in the region of Breda (i.e., municipality region of Breda [GM0758, PV30]: Bavel, Breda, Prinsenbeek, Teteringen, Ulvenhout) will be invited to participate. This region has been selected because it is more or less representative of the Dutch situation (urban center, with rural surroundings) and has not been subjected to screening efforts in the past (as has been done for instance in Groningen with the PREVEND [an observational albuminuria based cohort study previously performed in the city of Groningen by our research group (6)] and the Lifelines study). This age range has been selected because it is expected that screening in such an age range is probably the most cost-effective (9, 10) (we will also examine the most cost-effective age range within this range, see paragraph 9.2). Before the start of the study, all general practitioners (GPs) in this region will be informed about the study. Subjects will be selected to be representative for the Dutch general population (with respect to sex distribution, age distribution and socio-economic status with the use of data of Statistics Netherlands [Centraal Bureau voor de Statistiek]). In this study, we will investigate two different screening techniques for albuminuria screening. All subjects will be 1:1 randomized stratified for sex, age and socio-economic status into approach A (screening of albuminuria with the PeeSpot test; N=7,516) or approach B (screening of albuminuria with the ACR | EU test; N=7,516).

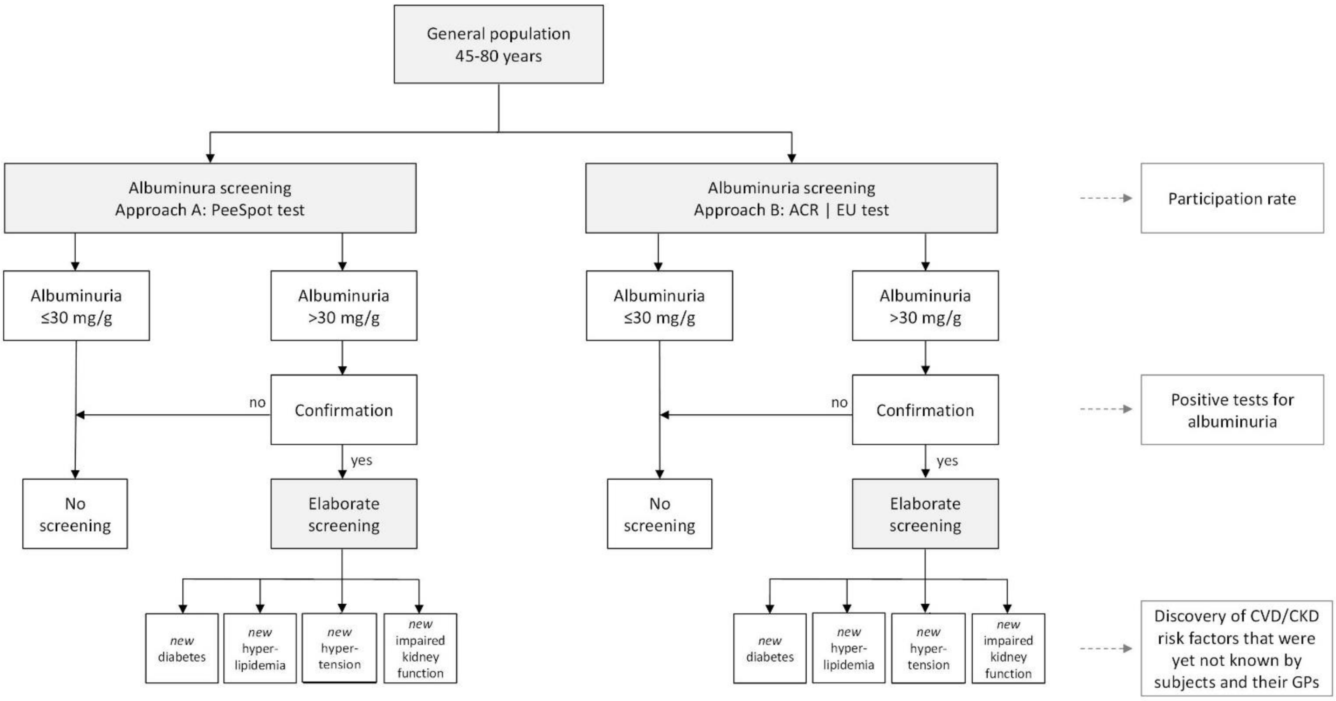

Figure 2. Schematic presentation of the study.

All individuals will receive an invitation to participate via mail (see Appendix E1.1 Proefpersoneninformatie groep A (PeeSpot methode) [versie 4.2, 23-07-2019], E1.2 Proefpersoneninformatie groep B (ACR EU methode) [versie 5.0, 03-12-2020], E2.5 Informatiefolder groep A (PeeSpot methode) [versie 3.0, 03-12-2019] and E2.6 Informatiefolder groep B (ACR EU methode) [versie 3.0, 03-12-2019]), including information about the study, the allocated test (either PeeSpot urine collection device or ACR | EU test), and instructions for the allocated test (see Appendix E2.1 Gebruiksaanwijzing groep A (PeeSpot methode) [versie 5.0, 03-12-2019] and E2.3 Test instructies ACR EU [versie 5.0, 03-12-2019]). All subjects for the albuminuria screening will be invited in tranches.

Approach A
Participants randomized to approach A will receive the PeeSpot urine collection device (Hessels+Grob B.V., Deventer, The Netherlands (11)). For a more detailed description of this device see Chapter 6. In brief, the PeeSpot urine collection device consists of a holder containing a urine collection pad. When subjects urinate on the pad (midstream, early morning void), it absorbs 1.2 mL urine. The participant can place the holder back into the tube, which can be easily send to the laboratory by mail for measurement of albuminuria. Subjects will, together with the invitation, receive an informed consent form via mail which subjects can include in the return envelope for the PeeSpot urine collection device to the laboratory. In the laboratory, the urine collecting device can be centrifuged to separate the urine from the pad, and urinary albumin and creatinine concentrations can be measured for assessment of the ACR. The results of the test will be sent to the participant by a letter via mail. If the test result is negative, there is no need for further examinations. If the test result is positive (ACR >30 mg/g), which indicates elevated albuminuria, participants will be sent an extra PeeSpot urine collection device for confirmation. When the test result of the extra test is normal (which indicates no elevated albuminuria), participants will be send a third test. When elevated albuminuria is confirmed (either by the second or the third test, at least by 1 confirmatory test in accordance with prevailing guidelines), the participant will be invited for further screening for CKD and CVD risk factors at a central facility (see paragraph ‘Elaborate Screening’ below).

Approach B
Participants randomized to approach B will receive the ACR | EU test (Healthy.io Ltd, Tel Aviv-Yafo, Israel). For a detailed description of the test see Chapter 5. In brief, the ACR | EU test kit consists of a urine test strip, a urine cup, a color calibrator, and instructions to download a smartphone application. First, subjects have to download the application on their smartphone and via the app they can complete an informed consent form. For the test, subjects have to collect urine in the cup (midstream, early morning void) and immerse and remove the urine test strip of which the chemical pads will change in color in response to the presence of albuminuria and creatinine. The stick is placed on the color calibrator and scanned with the application with the use of the flash light. Participants then enter the unique kit ID (which is printed on the kit) into the app for pseudonymous identification. Healthy.io’s system automatically analyzes the semi-quantitative albumin and creatinine levels indicated

on the stick and determines an ACR category (normal, abnormal, high abnormal) following the KDIGO A1, A2, A3 categorization (2). The test result is directly shown to the participant in the app. If this initial test result is negative, there is no need for further examinations. If the test result is positive (ACR >30 mg/g), which indicates elevated albuminuria, participants will be sent an extra ACR | EU test for confirmation. When the test result of the extra test is normal (which indicates no elevated albuminuria), participants will be send a third test. When elevated albuminuria is confirmed (either by the second or third test, at least by 1 confirmatory test in accordance with prevailing guidelines), the participant will be invited for further screening for CKD and CVD risk factors at a central facility (see paragraph ‘Elaborate Screening’ below).

Maybe positive subset

We will invite subjects of group A (PeeSpot group) with an ACR between 20 and 30 mg/g for a second test (estimated to be 2-3% of the responders from group A). If the ACR of this second test is again <30 mg/g, this is the end of the study for the participant. If the ACR of the second test is ≥30 mg/g, we will invite the participant to do a third test. If this third test is <30 mg/g, this is the end of the study for this participant (2 out of 3 negative). Is the third test is ≥30 mg/g, the subject will be invited for the elaborate screening in the Amphia hospital (ACR in 2 out of 3 test ≥30 mg/g). We do this, because it is not known yet what the best cut-off value of the ACR is in (pre-)screening. It has been suggested that lowering the cut-off value for the pre-screening might render better results i.e. higher yield of the screening (19). With collecting these data, we are able to evaluate the best cut-off value of ACR to achieve the highest yield of the screening.

False-negative subset

Furthermore, we will invite a randomly selected subset of 1000 participants (500 for each screening technique) who had a negative first test to perform an extra tests (with the PeeSpot urine collection device as this provides a quantitative measure of the ACR) to examine the false-negative result rate for both screening methods. When the extra test is also negative (indicating no elevated albuminuria), there is no need for further examinations. In case the extra test is positive, a third test will be send to confirm the result. If the third test is also positive (indicating elevated albuminuria), the participant will be invited to participate in the elaborate screening at a central facility (see paragraph ‘Elaborate Screening’ below).

At the end of the screening period, the research team will contact the GPs and the pharmacies of the participants who were selected in this random subset and who had a first false negative test, to check whether the participant has a disease or is/was using medication which can alter albuminuria levels.

Elaborate screening

Subjects with confirmed elevated albuminuria (at least 2 out of the 2 or 3 tests positive) will be invited for further screening for CKD and CVD risk factors, including hypertension, diabetes, hypercholesterolemia, and impaired kidney function, in the central screening facility located at the Amphia Hospital, Breda. An additional informed consent will be requested for

participating this elaborate screening. During this visit, anthropometric measurements will be performed (height and weight), blood pressure will be measured, and blood will be drawn (venipuncture, 7 mL) for assessing the traditional risk factors for CVD and CKD (including HbA1c, (non-) fasting glucose, total cholesterol, high-density lipoprotein (HDL) cholesterol, low density lipoprotein (LDL; as determined by Friedewald formula (12); note: if triglyceride level >4.52 mmol/L, LDL cholesterol cannot be determined) cholesterol, triglycerides, and creatinine). A novel ACR will be determined in a spot urine sample collected at the screening facility. The time of the urine sample, as well as the fasting state (not fasting, fasting no food, fasting no water and no food) will be reported during the screening.

Based on the results of the elaborate screening (i.e. when a risk factor is found, that was previously undiagnosed, and that needs treatment) subjects will be referred to their GP to be prescribed lifestyle measures and/or medical intervention according to prevailing Dutch guidelines (NHG Standards for Cardiovascular Risk Management and CKD). If no abnormalities are found (next to the albuminuria), the participant will receive a letter with all results and a recommendation to visit their GP after 1 year, to again screen for all risk factors according to the prevailing guidelines. The results of the screening and the recommendation to screen the participant again after 1 year will also be send to the GP (contact details of GP will be asked in the questionnaire, see paragraph ‘Questionnaire’ below). In case the participant was already known with the present risk factor (this will be asked in a questionnaire, see paragraph ‘Questionnaire’ below), but appear poorly controlled and/or high albuminuria status was yet undiagnosed which may need a change in treatment, subjects will also be referred to their GP to achieve better control of risk factors, again according to aforementioned prevailing Dutch guidelines. The recommendation to visit the GP will be accompanied by a letter with all results of the screening. This information will also be sent to the GP. At the end of the screening period, the research team will contact the GPs and the pharmacies of the participants who were referred to their GP to check whether the participants did visit their GP, and if so, whether they started or changed treatment (lifestyle and/or medication) of cardiovascular- or renal risk factors, including hypertension, hypercholesterolemia, diabetes, and impaired renal function.

False negative subset invited to elaborate screening

In total, 200 subjects from group A and 200 subjects from group B who are selected for the false-negative subset (first test ACR <30 mg/g) with a second negative test, will be invited to participate the elaborate screening. In that way, we can examine the differences in rate of previously undiagnosed risk factors (hypertension, diabetes, hypercholesterolemia, and impaired kidney function) that were found at the elaborate screening between subjects who have albuminuria and subjects without albuminuria.

Questionnaire

A subset of participants will receive a questionnaire (see Appendix ‘F1. Vragenlijst’ for the complete questionnaire). This subset of participants include: 1) all subjects with an overall positive test for albuminuria (at least 2 out of 2 or 3 ACR tests positive) invited to the elaborate screening,2) a subset of subjects with an initial negative ACR test who will be

asked to perform a second and third test to examine the false-negative rates (N=300 for both arms), and 3) a subset of the false-negative subset with a second negative test that will be invited for the elaborate screening (N=200 for both arms). The questionnaire includes questions regarding:

1) Demographic variables (age, sex, height, weight, ethnic background [the latter is needed to accurately estimate the glomerular filtration rate as measure of renal function and to investigate whether several ethnic subgroups have a higher CKD/CVD risk (13)], educational level), disease (including the presence/history of increased albuminuria, hypertension, diabetes, hypercholesterolemia, cardiovascular disease, and decreased renal function), use of medication;
2) Quality of life, the EuroQol 5D-5L (EQ-5D): a standardized questionnaire that provides a score at five health levels (mobility, self-care, daily activities, pain/discomfort, and anxiety/depression), whereby a weighted health index can be derived for an individual (information that is needed for cost-effectiveness studies);
3) Usability of the screening method (based on user preference, satisfaction rank, percentage that described the process as easy, and usability success);
4) All Aspects of Health Literacy Scale (AAHLS) questionnaire, and;
4) Subjects invited to the elaborate screening and the randomly selected subset of 1000 participants will also receive a question to provide the contact details of their GP and pharmacy. Moreover, these participants will receive an additional informed consent which will request:
   - to give permission to contact their GP to send the results of the screening;
   - to give permission to contact their GP/pharmacy to request data on treatment after the elaborate screening, and;
   - to give permission to store the leftover blood and urine samples at -80°C for possible further analyses in line with the present study.

Reminders

For both screening methods, a written reminder will be sent to the invited subjects via mail in case they did not perform the first test within 3 weeks after sending the test. After 6 weeks sending the first test, a second written reminder will be sent to all invited participants who did not perform the test yet by that time. The same holds for the execution of the confirmatory tests and the elaborate screening. The time for adherence —the time between invitation and returning the test— will be unrestricted. Time for adherence will be only restricted by closing of the study (estimated to be in February 2020).

We will send the non-responders of the first 1,250 invited subjects of both groups (2,500 participants in total) a short questionnaire with questions regarding the reason(s) for not participating. We do this because we can evaluate whether there are any unclarities in the information material or test instructions so we can change this, or if the non-responders just do not want to participate. For completing and returning this short questionnaire, participants will receive a ‘VVV-bon’ with the value of €10,00. We will do this to increase the response of this group.

4. STUDY POPULATION

4.1 Population (base)

A random sample of 15.032 subjects will be drawn from the population aged 45-80 years from the region of Breda (i.e., municipality region of Breda [GM0758, PV30]: Bavel, Breda, Prinsenbeek, Teteringen, Ulvenhout) by Statistics Netherlands (CBS). The "Rijksdienst voor Identiteitsgegevens" (Dutch Ministry of the Interior and Kingdom Relations) will provide us with the following data of the random sample: first name, middle name, last name, date of birth, sex, and address (including street name, house number, postal code, and town). They will deliver the requested data in batches (once every month) to keep the data up to date with respect to moving and death. This random sample, will be representative for the Dutch population with respect to age distribution, sex distribution, and estimated socioeconomic class based on postal code areas, see Figure 3. This Figure shows that the age and sex distribution of the Netherlands (panel A) and of Breda (panel C) are comparable. Moreover, the estimated socioeconomic status of the subjects aged 45-80 years living in the Netherlands based on status scores (presented in panel B) corresponds to that of subjects aged 45-80 years living in the region of Breda (panel D).

All subjects will be 1:1 randomized into approach A (receiving the PeeSpot collection device) or to approach B (receiving the ACR | EU test). Randomization will occur prior to invitation. Randomization will be done stratified for age, sex, and socioeconomic status (the latter will be based on data of Statistics Netherlands, providing average socioeconomic status per postal code area). Subjects living in one household will be randomized to the same group.

Date of birth is requested from the "Rijksdienst voor Identiteitsgegevens", as this information is needed for the communication of the results with the GPs and for retrieving data with respect to death and cause of death from Statistics Netherlands (CBS). The region of Breda is chosen because no other screening project –including albuminuria screening– has previously been performed in this region and because of the combination of rural and urban area which is approximately representative for the Dutch population. The age range 45-80 has been selected because it is expected that screening in such an age range is suggested to be the most cost-effective (9, 10).

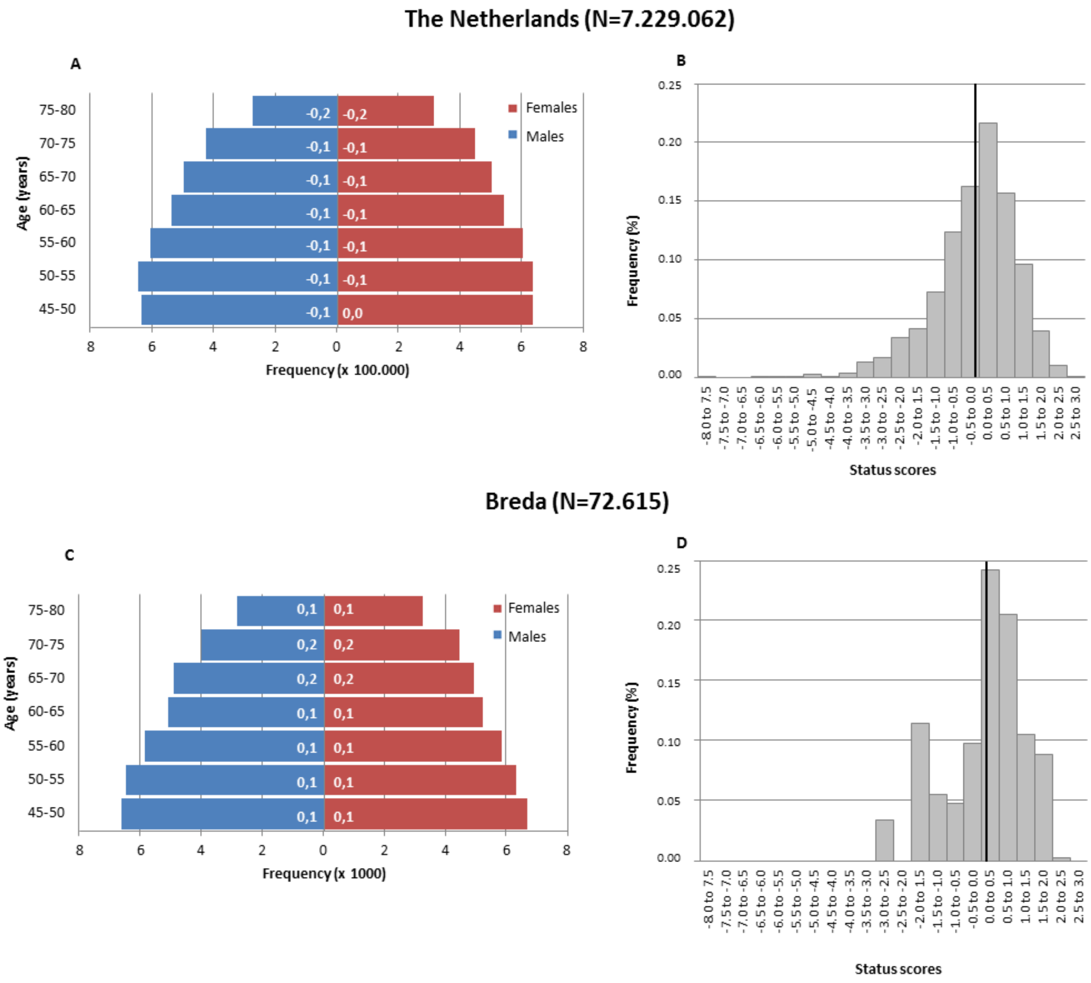

Figure 3. Distribution of age, sex and socioeconomic status of subjects aged 45-80 living in the Netherlands (panel A and B) and in the region of Breda (panel C and D). The age and sex distribution of the Netherlands is presented in panel A and of Breda in panel C. The white numbers shown in panels A and C represent the mean status scores of that specific age category. The estimated socioeconomic status of the subjects aged 45-80 years living in the Netherlands based on status scores is presented in panel B, whereas the estimated socioeconomic status based on status scores of subjects aged 45-80 years living in the region of Breda is presented in panel D. The black lines in panels B and D represent the mean status score of that population. Data on status scores are derived from Sociaal en Cultureel Planbureau of the Netherlands (from url: https://www.scp.nl/Onderzoek/Lopend_onderzoek/A_Z_alle_lopende_onderzoeken/Statusscores, accessed on 07-03-2019). The social status of a neighborhood (=status score) is derived from a number of characteristics of the people who live there: their education, income and position on the labor market. Data on age- and sex distribution was derived from Statistics Netherlands (from url: https://www.cbs.nl/nl-nl/maatwerk/2018/49/bevolking-en-huishoudens-4-cijferige-postcode-1-1-2018, accessed on 07-03-2019).

4.2 Inclusion criteria

In order to be eligible to participate in this study, a subject must meet all of the following criteria: aged 45-80 years and living in the region of Breda (i.e., municipality region of Breda [GM0758, PV30]: Bavel, Breda, Prinsenbeek, Teteringen, Ulvenhout).

4.3 Exclusion criteria

In order to be eligible to participate in this study, a subject will be excluded when the following criteria applies: younger than 45 years, older than 80 years, not living in the region of Breda (i.e., municipality region of Breda [GM0758, PV30]: Bavel, Breda, Prinsenbeek, Teteringen, Ulvenhout), or when institutionalized.

4.4 Sample size calculation

The primary outcome measurements will be the participation rate and yield of the screening (i.e. number of subjects with elevated albuminuria with newly diagnosed CKD and CVD risk factors including hypertension, diabetes, hypercholesterolemia, and impaired kidney function) and the difference of the prevalence of the yield of the screening between the two screening methods (PeeSpot versus ACR | EU). For the current study, we will need 15.032 subjects in total (7.516 in both arms). This sample size was calculated based on comparing two proportions with the following formula:

$$n = (Z\alpha/2+Z\beta)2 * (p1(1-p1)+p2(1-p2)) / (p1-p2)2$$

Where $Z\alpha/2$ is the critical value of the normal distribution at $\alpha/2$ (for a confidence level of 95%, α is 0.05 and the critical value is 1.96), $Z\beta$ is the critical value of the normal distribution at β (for a power of 80%, β is 0.2 and the critical value is 0.84) and p1 and p2 are the expected sample proportions of the two groups.

Based on experience from the PREVEND study (an observational albuminuria based cohort study previously performed in the city of Groningen by our research group (6)), we assume an overall participation rate of 50% (47.5% versus 52.5% for approach A and B, respectively), a first positive test for albuminuria in 8% of subjects, of which 90% will be confirmed by the second and/or third test (due to e.g., biological variation, not performing a second and/or third test), a participation rate in the elaborate screening of 90%.

Thus, inviting 15.032 subjects in total allows sufficient power (two-sided alpha 0.05 and beta 80%) to detect a 5% difference in participation rate between the two groups, which is deemed by us to be clinically relevant (i.e., 47.5% for approach A versus 52.5% for approach B). This number of subjects to be invited also allows us to (two-sided alpha of 0.05 and a beta of 80%) to discern a significant difference between both study arms in the number of individuals diagnosed with at least one newly discovered risk factor: the proportion of the overall population of 2.18 versus 1.73, i.e., a 71% chance to find a subject with at least one unknown risk factor in one group versus 51% in the other:

Approach A: 47.5% [participation rate] * 8% [proportion with albuminuria in first test] * 90% [proportion with positive confirmatory tests] * 90% [proportion participating elaborate screening] * 0.71 [chance to find a subject with at least one unknown risk factor]

and

Approach B: 52.5% [participation rate] * 8% [proportion with albuminuria in first test] * 90% [proportion with positive confirmatory tests] * 90% [proportion participating elaborate screening] * 0.51 [chance to find a subject with at least one unknown risk factor].

5. INVESTIGATIONAL PRODUCT

5.1 Name and description of investigational product

Participants randomized to approach B will receive the ACR | EU kit (Healthy.io Ltd, Tel Aviv-Yafo, Israel). This in-vitro diagnostic self-testing device with smartphone application for urine testing received the CE certificate in June 2018 (registration number D1417700004, report number P18-00200-114209). Figure 4 shows an example of a kit. The ACR | EU kit consists of instructions to download the app on a smartphone (1), a custom designed cup to collect urine (2), a urine dipstick (3), and a color board (4). The ACR | EU is CE and ISO 13485 certified for sale in the EU.

See Appendix ‘E2.3 Test instructies ACR EU [versie 3.0, 27-03-2019]’ for the test instructions that will be sent to the participants. The participants have to download the smartphone application according to the instructions included in the kit. The app guides the user through the testing process step-by-step. Subjects have to collect urine in the cup (midstream urine, early morning void) and immerse and remove the urine test strip of which the chemical pads will change in color in response to the presence of albuminuria and creatinine. The stick has to be placed on the color board and after 60 seconds after immersing in the urine (app will track the time), the stick can be scanned with use of the flash light. Participants then enter the unique kit ID (which is printed on each kit) into the app for pseudonymous identification. Healthy.io’s system measures albumin at a concentration between 10 mg/L-150 mg/L as well as creatinine 10-300 mg/dl and returns results for the ACR as a ‘normal’, moderately increased’ or ‘severely increased’ albumin levels following the KDIGO categorization: A1 (<30 mg/g), A2 (30-300 mg/g), A3 (>30

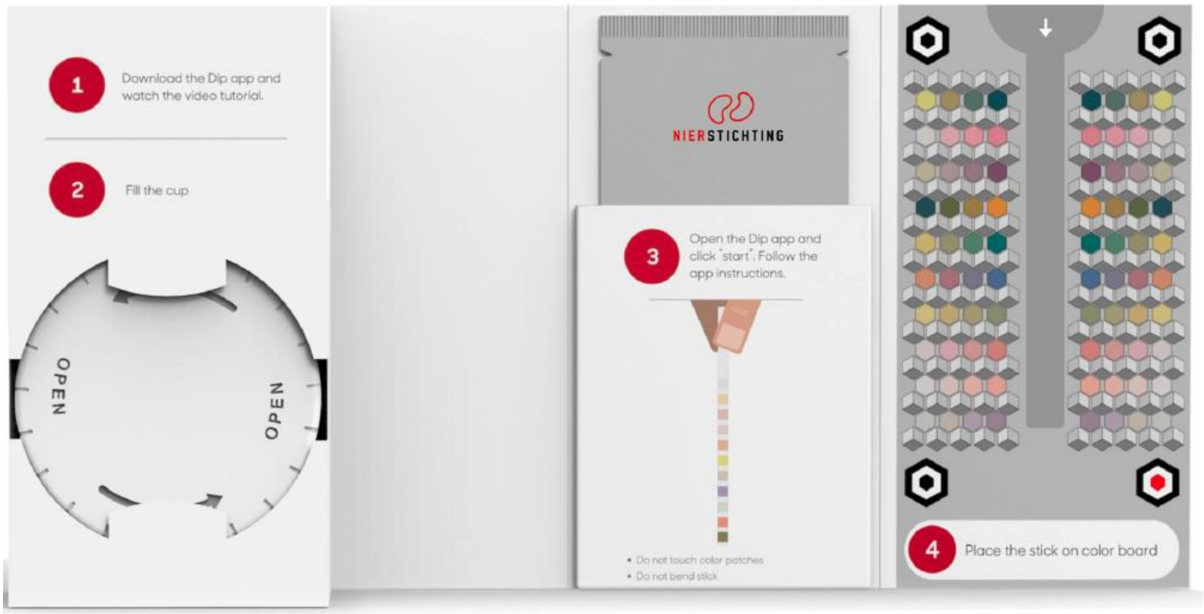

mg/g) (2).

Figure 4. Example of the ACR | EU test kit.

Results will be directly shown to the participant in the app. When the test is negative for increased albuminuria, participants will receive this result also in a letter via mail, together with the invitation to fill out the questionnaire as closing of the study. When the test is positive, participants will receive this result also in a letter via mail, together with the invitation to perform an extra ACR | EU test. See Appendix ‘E5.1 Brieven deelnemers [versie 5.0, 02-01-2020], Appendix ‘E2.3 Test instructies ACR EU [versie 3.0, 27-03-2019]’ for the instructions, and Appendix ‘D2.2 DAD ACR EU [versie 3.0, 04-04-2019]’ for product specification and risk classification.

5.2 Summary of findings from non-clinical studies

For information regarding the ACR | EU kit and its role in the diagnosis of albuminuria we refer to the Digitaal Aanschaf Dossier (Appendix ‘D2.2 DAD ACR EU [versie 3.0, 04-04-2019]’).

5.3 Summary of findings from clinical studies

For information regarding the ACR | EU kit and its role in the diagnosis of albuminuria we refer to the Digitaal Aanschaf Dossier (Appendix ‘D2.2 DAD ACR EU [versie 3.0, 04-04-2019]’).

5.4 Summary of known and potential risks and benefits

For information regarding the ACR | EU kit and its potential risks and benefits we refer to the Digitaal Aanschaf Dossier (Appendix ‘D2.2 DAD ACR EU [versie 3.0, 04-04-2019]’).

6. NON-INVESTIGATIONAL PRODUCT

6.1 Name and description of non-investigational product

The PeeSpot ® urine collection device is patented and registered by Hessels+Grob B.V.. See 'Appendix E2. Test instructies' for the test instructions that will be sent to the participants. It consists of a holder containing a urine absorption pad in a transport tube. The urine absorption pad is an absorption felt containing a dried hygroscopic polymer. For collecting a portion of urine with the PeeSpot, the absorbent pad is held in the urine stream for 3-5 seconds, in which approximately 1.2 ml of urine is absorbed. The holder can be placed back into the tube and can be easily sent to the laboratory by mail in a UN3373 envelop which will be sent together with the invitation letter, informed consent, and test kit. Because of the dried preservative in the urine absorption felt, the urine has a preservation capacity of 4 days at room temperature. In the laboratory, the PeeSpot tube can be centrifuged (5 minutes, 1800 G) and the urine will be released into the tube. In this urine, albumin, creatinine, and the ACR will be measured in the laboratory of the Amphia hospital.

6.2 Summary of findings from non-clinical studies

Not applicable.

6.3 Summary of findings from clinical studies

The results for various analytes determined in urine collected with the PeeSpot urine collection device or the same portion of urine collected routinely in a cup were compared using Altman-Bland bias plots and Passing and Bablock regression analysis (n=25). All chemical tests show excellent correlation between PeeSpot urine and a cup, both stored at 37 °C for 3 hours and at room temperature for 3 days, including the measurements of albuminuria and creatinine (both *r*=1.0) (11).

Furthermore a study in young, not continent children conclude that the PeeSpot is an accurate and precise tool for collecting urine for albumin measurement in young children and should be preferred over the alternative cotton wool collection technique (14).

6.4 Summary of known and potential risks and benefits

No risks are expected for the user. For information regarding the PeeSpot and its potential risks and benefits we refer to the Digitaal Aanschaf Dossier (Appendix 'D2.1 DAD PeeSpot [versie 1.0, 04-04-2019]').

Only potential risks apply to the quality of the urine sample collected with the PeeSpot, as this might induce extra variability in the albumin concentration with low and high temperatures. Extreme weather conditions could therefore be a potential risk. To avoid this risk, in such weather conditions (temperatures outside above 25 °C or below 0 °C), we will ask the participant not to return the envelope in an outside PostNL mailbox, but to bring the envelope to a post office or parcel service point, or to wait with urine collection and sending to less extreme weather conditions. If there will be such extreme

weather conditions, we will also temporarily stop inviting subjects. Of note, in previous studies it has been shown that frozen storage of urine at temperatures between freezing at 0 and -20 °C results in extra variability in measurement of albumin concentration (15).

Another issue which might introduce more variability in the urine albumin concentrations is the risk that the participant does not directly send the urine sample to the laboratory the same day as the test has been performed. Due to the dried preservative in the urine absorption felt, the urine has a preservation capacity of 4 days at room temperature. We will therefore give the participant clear instructions to send the test back to the laboratory the same day as the test is performed. Furthermore, we will also ask the participant to not perform and send the test on Fridays, as the test will then arrive in the laboratory not earlier then the following Tuesday. A checklist will be included in the UN3373 return envelope on which the participants have to write down the date on which the urine sample was taken. On this same checklist, participants have to declare that they are not: having fever, are menstruating, are pregnant, or have a urinary tract infection at day the urine sample was taken (this is also included in the instructions).

6.5 Description and justification of route of administration and dosage

Not applicable.

6.6 Dosages, dosage modifications and method of administration

Not applicable.

6.7 Preparation and labelling of Non Investigational Medicinal Product

Not applicable.

6.8 Drug accountability

Not applicable.

7. METHODS

7.1 Study parameters

7.1.1 Main study parameters

Participation rate of the two screening methods (defined as the number of persons completing the albuminuria screening (i.e. returning the first PeeSpot urine device or scanned the first ACR | EU urine test strip with use of the app, and in case of an ACR >30 mg/g in this initial test, also completing the two additional albuminuria screening tests), the positivity rate of the ACR tests (defined as the number of persons who tested positive for albuminuria (at least 2 out of 2 or 3 tests positive) relative to the number of persons participating in the corresponding arm (=per-protocol analysis) and of all invited persons in the corresponding arm (intention-to-screen analysis), and the positivity rate of the elaborate screening, defined as the number of subjects with increased albuminuria (defined as ACR >30 mg/g) with newly diagnosed and/or poorly controlled CVD and CKD risk factors. These risk factors, which will be assessed during the elaborate screening, include:

- Hypertension, defined as a systolic blood pressure of ≥130 mm Hg, a diastolic blood pressure of ≥80 mm Hg, and/or the use of antihypertensive drugs (in accordance with the KDIGO Guideline for CKD (16));
- Diabetes Mellitus, defined as fasting plasma glucose ≥7.0 mmol/L (>126 mg/dL), a non-fasting plasma glucose ≥11.2 mmol/L, HbA1c ≥6.5% (48 mmol/mol), and/or the use of glucose-lowering medication (in accordance with the prevailing Dutch guideline NHG Standard Diabetes mellitus type 2);
- Unfavourable lipid profile, defined as total cholesterol >5 mmol/l, low-density lipoprotein (LDL) cholesterol >2.6 mmol/L), triglycerides >2 (fasting 1.7), high-density lipoprotein (HDL) cholesterol <1, and/or the use of lipid lowering medication (in accordance with the prevailing Dutch guideline NHG Standard for Cardiovascular Risk Management);
- Impaired renal function, defined as an estimated glomerular filtration rate (eGFR, with the use of the creatinine-based Chronic Kidney Disease Epidemiology Collaboration, taking into account age, sex, and race (17)) of <60 ml/min/1.73 m^2 (in accordance with the KDIGO Guideline for CKD (18));

7.1.2 Secondary study parameters

- Completion rate of the study, defined as the number of persons completing the study relative to the number of all invited persons in the corresponding arm (intention-to-screen). Completion of the study includes: 1) completing the albuminuria screening (i.e. returning the first PeeSpot urine device or scanned the first ACR | EU urine test strip with use of the app, 2) in case of an ACR >30 mg/g in this initial test, also completing the required additional albuminuria screening

test[s]), 3) and when invited to the elaborate screening when tested positive overall (at least two out of two or three tests positive) also completing the elaborate screening in the Amphia Hospital;

- Completion and GP follow-up rate study, defined as the number of persons completing the study (ACR testing, elaborate screening when invited, and visiting GP when recommended) relative to the number of all invited persons in the corresponding arm (intention-to-screen). Completion of the study including GP follow-up includes: 1) completing the albuminuria screening (i.e. returning the first PeeSpot urine device or scanned the first ACR | EU urine test strip with use of the app, 2) in case of an ACR >30 mg/g in this initial test, also completing the required additional albuminuria screening test[s]), 3) when invited to the elaborate screening when tested positive overall (at least two out of two or three tests positive) also completing the elaborate screening in the Amphia Hospital, and 4) when abnormalities are found in the elaborate screening, also visiting their GP;
- Information on (differences in) characteristics of the responders of the two screening methods (PeeSpot vs. ACR | EU) including differences in age, sex, educational level, estimated social economic status (based on data of Statistics Netherlands, providing estimated social economic status based on postal codes), medication use, and history of disease;
- Information on (differences in) characteristics of the non-responders of the two screening methods (PeeSpot vs. ACR | EU) including differences in age, sex, and estimated social economic status;
- Information on rate of false-positive and -negative tests for both screening methods (PeeSpot vs. ACR | EU);
- Incremental cost-effectiveness ratio (ICER) in euro per QALY gained for the two screening methods;
- Usability scores of the two screening methods:
    - User preference: does the participant prefer doing this urine test (either PeeSpot or ACR | EU test) at home or does the participant prefer a standard urine test (going to the doctor's office to bring a sample of urine for testing).
    - Satisfaction rank: how likely is it that the participant would recommend this test to others for urine testing (5-points Likert scale question: very likely / likely / neutral / unlikely / very unlikely).
    - Percentage that described the process as easy (5-points Likert scale question: very easy / easy / neutral / difficult / very difficult).
    - Usability success: measured as share of people that tried to do a test that actually succeeded.
- The best ACR cut-off value to achieve the highest yield of the screening.
- The difference in in rate of previously undiagnosed risk factors (hypertension, diabetes, hypercholesterolemia, and impaired kidney function) that were found at the elaborate screening between subjects who have albuminuria and subjects without albuminuria.

7.1.3 Other study parameters

Contact details on participants' GP and pharmacy, data on whether the participant did visit their GP after elaborates screening when recommended based on the results of the screening, and if so, whether they started or changed treatment (lifestyle and/or medication).

7.2 Randomisation, blinding and treatment allocation

Participants will be randomized 1:1 to either approach A, the use of the PeeSpot urine collection device, or approach B, the use of the ACR | EU test. Randomization will be done by stratified for age, sex, and socioeconomic status (this latter will be based on data of Statistics Netherlands, providing average socioeconomic status per postal code area). In case per household two or more subjects will be invited to participate, these subjects will all be randomized to the same group.

7.3 Study procedures

The study procedures are summarized in Table 1. Procedures are dependent on the results of the albuminuria tests. A schematic presentation of the study is shown in Figure 2, Chapter 2.

Laboratory tests

Subjects randomized to group A (PeeSpot urine collection device) collect a urine sample with the PeeSpot urine collection device (see Chapter 6) and send this device to the laboratory of the Amphia Hospital for assessment of urine creatinine and albumin concentrations. Urine albumin concentration will be measured by immunotubidimetry and creatinine by specific enzymatic assay. The ACR (in mg/g) will be derived by dividing the urine albumin concentration with the urine creatinine concentration. In case increased albuminuria is found, the subject will receive an additional PeeSpot urine collection devices at home, to collect a new urine samples for ACR measurement as described previously. When this second test is negative (indicating no elevated albuminuria), the participant will receive a third test to confirm this result. In case the second or third urine samples also tests positive for increased albuminuria, the overall ACR test will be called positive (at least 2 out of 2 or three positive) and these subjects will be invited for an elaborate screening in the Amphia Hospital, Breda.

Subjects randomized to group B (ACR | EU test) will test a urine sample at home as described in Chapter 5. In case the test is positive for increased albuminuria, the subject will receive an additional ACR | EU test. When this second test is negative (indicating no elevated albuminuria), the participant will receive a third test to confirm this result. In case the second or third extra urine samples also tests positive for increased albuminuria, the overall ACR test will be called positive (at least 2 out of 2 or three positive) and these subjects will be invited for an elaborate screening in the Amphia Hospital, Breda.

In subjects tested negative for albuminuria in the first test, no further laboratory tests will be performed, except for a random subset of 1000 subjects (500 in each group) with an initial negative ACR test. These 1000 subjects will receive an additional ACR test (PeeSpot test as this test provides an quantitative measure of the ACR) to investigate the rate of false-negative tests.

At the elaborate screening in the Amphia hospital, blood will be drawn (see paragraph below) for assessment of HbA1c, (non-)fasting glucose, total cholesterol, HDL cholesterol, LDL cholesterol (according to the Friedewald formula (12)), triglycerides, and creatinine. Furthermore, an additional spot urine sample will be collected for ACR measurement using standard lab technology as mentioned previously.

Based on data of the PREVEND study (a UMC Groningen observational study centered around albuminuria measurement (4)) we expect that around 8% of the participating subjects (expected participation rate is 50%, also based on results of the PREVEND study) will have a first positive ACR test. Of these, we expect that 90% of the subjects will be confirmed by a second and third ACR test. These subjects will be invited for the elaborate screening in the Amphia hospital (estimated to be N≈600 subjects).

	**First urine test**	**Confirmatory urine tests**	**Elaborate screening**
**(SPOT) URINE TESTS**			
Albumin concentration	X	X	X
Creatinine concentration	X	X	X
ACR	X	X	X
Biobanking left-over material			X
**BLOOD TESTS**			
HbA1c			X
Glucose			X
Total cholesterol			X
HDL cholesterol			X
LDL cholesterol*			X
Triglycerides			X
Creatinine			X
eGFR by CKD-EPI†			X
Biobanking left-over material			X
**PHYSICAL EXAMINATION**			
Blood pressure			X
Assessment of height and weight			X
**QUESTIONNAIRE**			
Demographic items disease, medication use		X‡	X
EQ-5D		X‡	X
Urine test usability		X‡	X

GP + pharmacy contact details		X‡	X

Table 1. Overview of the study procedures. Abbreviations: ACR, albumin-to-creatinine ratio; EQ-5D, EuroQol 5D-5L; GP, general practitioner; HDL, high-density lipoprotein; LDL, low-density lipoprotein.

* LDL will be determined by the Friedewald formula. Note: if triglyceride level >4.52 mmol/L, LDL cholesterol cannot be determined.

† eGFR will be calculated with the creatinine-based Chronic Kidney Disease Epidemiology Collaboration equation (CKD-EPI) from 2009, taking into account age, sex, and race (17).

‡ Questionnaire will be taken at the end of the study in a subset of participants for assessing the false-negative test rates (500 subjects per arm).

Invasive procedures

Subjects with an overall positive ACR test (at least 2 out of 2 or 3 ACR tests) (N≈600 subjects, see above) and subjects selected from the false-negative subset with a second negative test (N=200 for both arms) will be invited for an elaborate screening in the Amphia Hospital, Breda. At this screening, blood will be drawn once (venipuncture, 7 mL) for assessing traditional risk factors for CVD and CKD, including HbA1c, (non-)fasting glucose, total cholesterol, HDL cholesterol, LDL cholesterol (according to the Friedewald formula (12)), triglycerides, and creatinine. Furthermore, an additional spot urine sample will be collected for ACR measurement using standard lab technology as mentioned previously. On the day before this screening, subjects are asked not to perform any strenuous physical. Subjects do not have to fast before the visit, however, it will be reported at the screening whether subjects were fasting (no water and/or no food).

Physical examination

In subjects tested negative for albuminuria, no physical examinations will be performed. Subjects with overall positive ACR test (at least 2 out of 2 or 3 ACR tests) (N≈600 subjects, see above) will be invited for an elaborate screening in the Amphia Hospital, Breda. During this screening, blood pressure will be measured in five-fold on the right arm in sitting position with an automated device (the first two measurements will be discarded and the last three measurements will be averaged). Furthermore, anthropometric measurements will be performed (height and body weight without shoes and heavy clothing).

Questionnaire

A subset of participants will receive a questionnaire (see Appendix ‘F1. Vragenlijst’ for the complete questionnaire). This subset of participants include: 1) all subjects with an overall positive test for albuminuria (at least 2 out of 2 or 3 ACR tests positive) invited to the elaborate screening, 2) a subset of subjects with an initial negative ACR test who will be asked to perform a second (and if required a third) test to examine the false-negative rates (N=300 for both arms), and 3) a subset of the false-negative subset with a second negative test that will be invited for the elaborate screening (N=200 for both arms). The questionnaire includes questions regarding:

1) Demographic variables (age, sex, height, weight, ethnic background [the latter is needed to accurately estimate the glomerular filtration rate as measure of renal

function and to investigate whether several ethnic subgroups have a higher CKD/CVD risk (13)], educational level), disease (including the presence/history of increased albuminuria, hypertension, diabetes, hypercholesterolemia, cardiovascular disease, and decreased renal function), use of medication;

2) Quality of life, the EuroQol 5D-5L (EQ-5D): a standardized questionnaire that provides a score at five health levels (mobility, self-care, daily activities, pain/discomfort, and anxiety/depression), whereby a weighted health index can be derived for an individual (information that is needed for cost-effectiveness studies), and;
3) Usability of the screening method (based on user preference, satisfaction rank, percentage that described the process as easy, and usability success);
4) All Aspects of Health Literacy Scale (AAHLS) questionnaire, and;
5) Subjects invited to the elaborate screening and the randomly selected subset of 1000 participants will also receive the question to provide the contact details of their GP and pharmacy. Moreover, these participants will receive an additional informed consent which will request:
    - to give permission to contact their GP to send the results of the screening;
    - to give permission to contact their GP/pharmacy/medical specialists and research institutes (including Statistics Netherlands, Prismant) to request data on treatment and health information after the elaborate screening, and;
    - to give permission to store the leftover blood and urine samples at -80°C for possible further analyses in line with the present study.

Another subset (non-responders of the first 1,250 invited subjects of both groups, 2500 in total) will receive a short questionnaire with questions regarding the reason of non-response. See letters C1.A and C1B of document ‘E5.1 Brieven deelnemers [versie 5.0, 02-01-2019]’.

Referral to GP

Treatment is not included in the current study. Based on the abnormalities found in the elaborate screening, –i.e. when a risk factor is found, that was previously undiagnosed, and that needs treatment– subjects will be referred to their GP to be prescribed lifestyle measures and/or medical intervention according to prevailing Dutch guidelines (NHG Standards for Cardiovascular Risk Management and CKD). The results of the screening will also directly be sent to the GP (permission will be requested in an additional informed consent before the elaborate screening). In an accompanying letter, GP’s will also be asked to contact the patient to make an appointment in case the patient does not contact its GP.

In case the participant was already known with the present risk factor (this will be asked in the questionnaire), but the risk factor appeared poorly controlled and/or high albuminuria status was yet unknown making a change in treatment necessary, subjects will also be referred to their GP to achieve better control of risk factors, again according to aforementioned prevailing Dutch guidelines. The recommendation to visit their GP will be accompanied by a letter with all results of the screening. This information will also be sent

directly to the GP in case the participant gave permission to contact their GP (will be asked in questionnaire). In an accompanying letter, GP's will also be asked to contact the patient to make an appointment in case the patient does not contact its GP.

If no abnormalities are found in the elaborate screening (next to albuminuria), the participant will receive a letter with all results and a recommendation to visit their GP after 1 year, to again screen for all risk factors according to the prevailing guidelines. The results of the screening and the recommendation to screen the participant again after 1 year will also be send to the GP.

7.4 Withdrawal of individual subjects

Subjects can leave the study at any time for any reason if they wish to do so without any consequences.

7.5 Replacement of individual subjects after withdrawal

Only when the invitation letter will be sent back to us in case the invited person is not known at the address or has died in the meantime, we will invite another subject to participate.

7.6 Follow-up of withdrawn subjects

After withdrawal, no follow-up of subjects will take place.

7.7 Premature termination of the study

Premature termination of the study is not anticipated, as no harmful effects of the screening methods are expected that will jeopardise subjects health or safety. Very low or high participation rates are no reasons for premature termination of the study, as this is one of the primary outcomes we want to examine.

8. SAFETY REPORTING

8.1 Temporary halt for reasons of subject safety

Given the nature of the study, we do not expect that there will be reasons for a temporary halt for reason of subject safety. However, in accordance to section 10, subsection 4, of the WMO, the sponsor will suspend the study if there is sufficient ground that continuation of the study will jeopardise subject health or safety. The sponsor will notify the accredited METC without undue delay of a temporary halt including the reason for such an action. The study will be suspended pending a further positive decision by the accredited METC. The investigator will take care that all subjects are kept informed.

8.2 AEs, SAEs, and SADEs

8.2.1 Adverse events (AEs)

Adverse events (AEs) are defined as any undesirable experience occurring to a subject during the study, whether or not considered related to the (non) investigational product. Given the nature of the study, we do not expect that any AE will take place. However, in case a subject spontaneously report an AE or the investigator observes an AE, this will be recorded.

8.2.2 Serious adverse events (SAEs)

Given the nature of the study, we do not expect that any serious adverse event (SAE) will take place. An SAE is any untoward medical occurrence or effect that

- results in death;
- is life threatening (at the time of the event);
- requires hospitalisation or prolongation of existing inpatients' hospitalisation;
- results in persistent or significant disability or incapacity;
- is a congenital anomaly or birth defect; or
- any other important medical event that did not result in any of the outcomes listed above due to medical or surgical intervention but could have been based upon appropriate judgement by the investigator.

An elective hospital admission will not be considered as a serious adverse event.

In case a subject spontaneously report an SAE or the investigator observes an SAE, this will be recorded. In the unforeseen case that there is a SAE, the sponsor will report the SAE through the web portal *ToetsingOnline* to the accredited METC that approved the protocol, within 7 days of first knowledge for SAEs that result in death or are life threatening followed by a period of maximum of 8 days to complete the initial preliminary report. All other SAEs will be reported within a period of maximum 15 days after the sponsor has first knowledge of the serious adverse events.

8.2.3 Serious adverse device events (SADEs)

Given the nature of the study, we do not expect that any serious adverse device event (SADE) will take place. An SADE is any untoward medical occurrence or effect in research with a medical device that

- results in death;
- is life threatening (at the time of the event);
- requires hospitalisation or prolongation of existing inpatients' hospitalisation;
- results in persistent or significant disability or incapacity;
- is a congenital anomaly or birth defect; or
- any other important medical event that did not result in any of the outcomes listed above due to medical or surgical intervention but could have been based upon appropriate judgement by the investigator.

An elective hospital admission will not be considered as a serious adverse event.

In case a subject spontaneously report an SADE or the investigator observes an SADE, this will be recorded. In the unforeseen case that there is a SADE, the sponsor will report the SADE through the web portal *ToetsingOnline* to the accredited METC that approved the protocol, within 7 days of first knowledge for SADEs that result in death or are life threatening followed by a period of maximum of 8 days to complete the initial preliminary report. All other SADEs will be reported within a period of maximum 15 days after the sponsor has first knowledge of the serious adverse events.

8.3 Follow-up of adverse events

AEs, SAEs, and SADEs need to be reported till end of study within the Netherlands, as defined in the protocol.

9. STATISTICAL ANALYSIS

Data on baseline characteristics will be summarized for continuous variables, in case of normal distribution by mean and standard deviation, and in case of non-normal distribution by median and interquartile range. For discrete variables (e.g., sex and ethnic background) data will be summarized by number and percentages.

9.1 Primary study parameter(s)

Participation rate will be defined as the number of persons completing the albuminuria screening (i.e. returning the first PeeSpot urine device or scanned the first ACR | EU urine test strip with use of the app, and in case of an ACR >30 mg/g in this initial test, also completing the required additional albuminuria screening test[s]) relative to the number of all invited persons in the corresponding arm (intention-to-screen). Differences in proportions between the two screening arms will be calculated with the Chi-square test.

The positivity rate of the ACR tests will be calculated separately for the PeeSpot arm as the ACR | EU arm, as the number of persons who tested positive for albuminuria (at least 2 out of 2 or 3 tests positive) relative to the number of persons participating in the corresponding arm (=per-protocol analysis) and of all invited persons in the corresponding arm (intention-to-screen analysis). Differences in proportions between the two screening arms will be calculated with the Chi-square test.

The positivity rate for the elaborate screening will also be calculated separately for the PeeSpot arm as the ACR | EU arm. This will be calculated as the number of persons with a positive test for albuminuria (at least 2 out of 2 or 3 tests) and with a newly diagnosed CVD and/or CKD risk factor (i.e., hypertension, diabetes, hypercholesterolemia and/or impaired kidney function) relative to the number of persons participating the elaborate screening in the corresponding arm (=per-protocol analysis) and of all invited persons for the elaborate screening in the corresponding arm (intention-to-screen analysis). Differences in proportions of subjects with newly diagnosed risk factors between the two screening arms will be calculated with the Chi-square test.

9.2 Secondary study parameter(s)

Participation rates

Completion rate of the study will be defined as the number of persons completing the study relative to the number of all invited persons in the corresponding arm (intention-to-screen). Completion of the study includes: 1) completing the albuminuria screening (i.e. returning the first PeeSpot urine device or scanned the first ACR | EU urine test strip with use of the app, 2) in case of an ACR >30 mg/g in this initial test, also completing the required additional albuminuria screening test[s]), 3) and when invited to the elaborate screening when tested positive overall (at least two out of two or three tests positive) also

completing the elaborate screening in the Amphia Hospital. Differences in proportions between the two screening arms will be calculated with the Chi-square test.

Completion and GP follow-up rate study will be defined as the number of persons completing the study (ACR testing, elaborate screening when invited, and visiting GP when recommended) relative to the number of all invited persons in the corresponding arm (intention-to-screen). Completion of the study including GP follow-up includes: 1) completing the albuminuria screening (i.e. returning the first PeeSpot urine device or scanned the first ACR | EU urine test strip with use of the app, 2) in case of an ACR >30 mg/g in this initial test, also completing the required additional albuminuria screening test[s]), 3) when invited to the elaborate screening when tested positive overall (at least two out of two or three tests positive) also completing the elaborate screening in the Amphia Hospital, and 4) when abnormalities are found in the elaborate screening, also visiting their GP. Differences in proportions between the two screening arms will be calculated with the Chi-square test. Differences in rate of previously undiagnosed risk factors (hypertension, diabetes, hypercholesterolemia, and impaired kidney function) that were found at the elaborate screening between subjects who have albuminuria and subjects without albuminuria will be calculated with the Chi-square test.

Usability

The usability of the tests (PeeSpot and ACR | EU) will be assessed by different aspects:

1. User preference: does the participant prefer doing this urine test (either PeeSpot or ACR | EU test) at home or does the participant prefer a standard urine test (going to the doctor's office to bring a sample of urine for testing).
2. Satisfaction rank: how likely is it that the participant would recommend this test to other for urine testing (5-points Likert scale question: very likely / likely / neutral / unlikely / very unlikely), expressed as Net Promotor Score.
3. Percentage that described the process as easy (5-points Likert scale question: very easy / easy / neutral / difficult / very difficult).
4. Usability success: percentage of participants that tried to do a test that actually succeeded.

Differences in usability scores of the both screening test will be calculated with the Chi-square test.

Participant characteristics

Univariable and multivariable logistic regression models will be fitted to the data to determine differences in the characteristics of the participants of the two screening strategies. Multivariable logistic regression models will include among others age, sex, height, weight, educational level, disease history (including presence of albuminuria, hypertension, diabetes, hypercholesterolemia, and impaired kidney function), medication use, and health literacy. Moreover, differences in characteristics between the responders and non-responders per screening method will be analyzed, again using univariable and multivariable logistic regression models.

False-positive and false-negative tests

To reduce the possibility of type I errors (false-positive findings), in both screening arms, subjects who tested positive for albuminuria in the first test, have to perform an extra test for confirmation. In case at least 2 out of 2 or 3 (in accordance to prevailing guidelines) in total are positive, participants are regarded as tested positive for albuminuria. In case only the first out of three tests was positive, the positive finding is seen as false-positive finding. To assess the percentage of subjects with false-negative findings, in both screening arms a random subset of 500 subjects will be selected who had a first negative test and will be invited to perform an extra test. In case this second test is positive and also the third test they will be invited for is positive, the first negative test will be seen as false-negative test result. Differences in proportions of subjects with false-positive and false-negative results between the two screening techniques will also be evaluated with the Chi-square test.

Cost-effectiveness

We will model the effects of the two screening methods on incidence of both CKD and CVD complications and endpoints by comparing to standard of care, i.e. when subjects would not have participated in the screening project. A literature search will be performed to identify all available information regarding the cost-effectiveness of screening for CKD and CVD. Patients quality of life (expressed as utilities, which is needed for cost-effectiveness analyses) will be obtained from the EQ-5D questionnaires, as well as being supported by literature. The information will be used to calculate effectiveness, expressed in Quality Adjusted Life Years (QALYs) gained for both screening interventions. The incremental cost-effectiveness ratio (ICER) in euro per QALY gained will be calculated, using input parameters from the literature and from data derived from this study. To identify the parameters with the most influence on the outcome, univariate sensitivity analyses will be conducted. Additionally, probabilistic sensitivity analyses will be conducted to account for uncertainty in all parameters included in the model. Moreover, we will determine which scenario analyses need to be conducted, e.g. by varying the age of the screening population to assess whether there is a more favorable cost-effectiveness ratio possible. Also by varying the ACR cut-off value, we will assess whether there is a more favorable cost-effectiveness ratio possible. State-of-the-art probabilistic sensitivity analysis will be used to further analyze uncertainty, using relevant and plausible distributions for core parameters. Finally, we will assess the outcomes of both cost-effectiveness analyses in a threshold analysis, determining which screening technique is the most favorable at a ceiling ratio of, for example, €20.000/QALYs and what corresponding maximum screening costs are aligned with such a threshold.

9.3 Other study parameters

We will also compare the number of subjects who were referred to their GP after they participated in the elaborate screening (only for subjects who provided permission to contact their GP), and the number of subjects who actually received new or a change in treatment (lifestyle measures and/or medical intervention according to prevailing Dutch

guidelines [NHG Standards for Cardiovascular Risk Management and CKD]). These data will be requested 2 months after completion of the elaborate screening from the participants' GP and pharmacy (permission will be asked in the additional informed consent before the elaborate screening in the Amphia hospital).

9.4 Interim analysis

Premature termination of the study is not anticipated, as no harmful effects of the screening methods are expected that will jeopardise subjects health or safety. Therefore, no interim analysis is planned. Very low or high participation rates are no reasons for premature termination of the study, as this is one of the primary outcomes we want to examine.

10. ETHICAL CONSIDERATIONS

10.1 Regulation statement

This study will be conducted according to the principles of the Declaration of Helsinki (October, 2013) and in accordance with the Medical Research Involving Human Subjects Act (WMO). All participants will have the right to withdraw from the study at any time.

10.2 Recruitment and consent

The research team will obtain data on all inhabitants in the region of Breda (i.e., municipality region of Breda [GM0758, PV30]: Bavel, Breda, Prinsenbeek, Teteringen, Ulvenhout) aged 45-80 years via the "Rijksdienst voor Identiteitsgegevens" (Dutch Ministry of the Interior and Kingdom Relations). These data include first name, middle name, last name, sex, date of birth, and address (including street, number, postal code, and town). Of these subjects, a random sample of 15.032 will be selected, representative for the Dutch population (with regard to age distribution, sex distribution, and estimated socioeconomic status, based on data of Statistics Netherlands [CBS]).

See Appendices 'E1.1 Proefpersoneninformatie groep A (PeeSpot methode) [versie 4.2, 23-07-2019]' and 'E1.2 Proefpersoneninformatie groep B (ACR EU methode) [versie 5.0, 03-12-2019]' for the invitation letters and informed consent forms for both arms of the study. Subjects randomized to group A (PeeSpot urine collection device) will directly with the invitation and information letter, receive an informed consent form via mail. The participant has to include the informed consent form in the return envelope for the PeeSpot urine collection device which has to be sent to the laboratory of the Amphia Hospital. Subjects randomized to group B (ACR | EU test) can complete a digital informed consent in the smartphone app. This informed consent procedure is offered digital, because the essence of the ACR | EU test is that it is a test participants can do at home. If the consent will be a written informed consent, participants have to go to the mail box. This will limit the participation rate in this group. The digital informed consent procedure will be executed with help of the company Zynyo, which is a signing service provider. This service is compliant with the law on electronic signatures (Artikel 3:15a van het Burgerlijk Wetboek). This law states that a digital signature has the same legal validity as a handwritten signature, if the authentication method that has been used is sufficiently reliable. For the authentication, subjects first have to read the informed consent and agree with the conditions stated in the informed consent by filling in their full name and e-mail address (this is the first step of the 2-factor authentication) and then click on 'I agree'. After this, subjects can do the urine test according the instructions. When scanning the image, the QR-code which is included on the colour board will also be scanned. This code functions as the second step in de 2-factor authentication, because the researchers have already linked this code to the participants' name when sending out the kits. After scanning the colour board, the data (participants' name, e-mail address and QR-code) will be send to the Zynyo signing server. Here, a PDF-file will be created

and sealed. Zynyo will send these files to the research team in the UMCG and also directly to the participant will receive the result of the urine test.

For both screening methods, a written reminder will be sent to the invited subjects via mail in case they did not perform the first test within 3 weeks after sending the test. After 6 weeks sending the test, a second written reminder will be sent to all invited participants who did not perform the test yet by that time. The same holds for the execution of the confirmatory tests and the elaborate screening. The time for adherence —the time between invitation and returning the test— will be unrestricted. Time for adherence will be only restricted by closing of the study (which is estimated to be March 2020).

10.3 Benefits and risks assessment, group relatedness

In case subjects participate the elaborate screening when elevated albuminuria is found, risk factors for CVD and CKD that were either not yet known, or were insufficiently treated can be found, including hypertension, diabetes, hypercholesterolemia, impaired renal function. Subjects with these abnormalities will be referred to their GP for lifestyle advice and/or medication treatment according to evidence based prevailing guidelines. Benefits with respect to health are therefore anticipated (these actually form the rationale to perform this study). Such benefits will be more or less similar in both arms.

The risks associated with the study are low, but the burden may be confrontation with an unfavorable result, unnecessary anxiety in case of false-positive test results, and unwarranted reassurance in case of false-negative results.

10.4 Compensation for injury

The sponsor, i.e., the UMCG (also) has an insurance which is in accordance with the legal requirements in the Netherlands (Article 7 WMO). This insurance provides cover for damage to research subjects through injury or death caused by the study. The insurance applies to the damage that becomes apparent during the study or within 4 years after the end of the study.

11. ADMINISTRATIVE ASPECTS, MONITORING AND PUBLICATION

11.1 Handling and storage of data and documents

A unique participant number will be assigned to all participants and will not change during the study. This number is linked with the name and address of the participant in a password-protected data platform (separated from the other data collected in the study). The principal investigator, the study coordinator, and three of the sub-investigators will have access to this key, amongst others for communication purposes (e.g., sending invitation letters and result letters) with the participants and GPs. All study data (ACR results, screening results, questionnaire results etc.) will be securely stored in a separate password-protected data platform.

E-Zorg B.V. is responsible for the data transfer of: 1) the ACR results from the Peespot measured in the laboratory of the Amphia hospital to the data platform, 2) the ACR | EU results from the app to the data platform, and 3) results of the elaborate screening in the Amphia hospital to data platform. Both data platforms will be hosted by a sub-processor of E-Zorg, i.e. Copernicus Interchange Technology. Data processing agreements which are compliant with the General Data Protection Regulation (GDPR) are set up and will signed by all parties involved in data processing.

All source documents pertaining to this study will be maintained by the investigators and made available for direct inspection by authorized persons. After 5 and 10 years after finishing the data collection of the study, data will be linked to data of Statistics Netherlands regarding date and cause of death. Fifteen years after the last linkage with Statistics Netherlands, the data will destroyed. Subjects will be asked permission and have to agree with this procedure, otherwise they cannot participate.

11.2 Monitoring and Quality Assurance

Monitoring will be executed in compliance with the NFU (The Netherlands Federation of University Medical Centers) guideline “Quality Assurance of research involving human subjects 2019”. Monitoring for this study will be performed by an independent and qualified monitor.

To ensure participants’ rights, wellbeing and safety, compliance as well as quality of data, the monitor will visit the site on a regular basis. For this study the risk classification is considered negligible (based on the NFU guideline), which implies monitoring of at least once per year. Because it is expected that the present study is only ongoing for 5-6 months, monitoring will take place before the start of the study (start is when participants will be invited) and 2 months after the start of the study.

The monitor will verify the participant flow (check of procedures for data transfer, informed consent and platform) before the start of the study. The monitor will verify the following items 2 months after the start of the study: Informed Consent (procedure and presence);

Trial Master File (presence of all essential documents); and reported SAEs (spontaneously reported by participant or observed by investigator). Source data verification will be performed for 0,5-1% of all participants (focused on informed consent form, inclusion criteria and reported SAEs/SADEs).

The presence of certificates, Standard Operating Procedures, and instructions related to devices, facilities, laboratories, and other departments involved will be checked. Findings from the monitoring visits will be reported by the monitor to the sponsor-investigator through a monitoring visit report. It is the responsibility of the sponsor-investigator to follow up on findings, deviations, queries, or other issues where required.

11.3 Amendments

Amendments are changes made to the research after a favourable opinion by the accredited METC has been given. All amendments will be notified to the METC that gave a favourable opinion.

11.4 Annual progress report

The sponsor/investigator will submit a summary of the progress of the study to the accredited METC once a year. Information will be provided on the date of inclusion of the first subject, numbers of subjects included and numbers of subjects that have completed the trial, serious adverse events/ serious adverse reactions, other problems, and amendments.

11.5 Temporary halt and (prematurely) end of study report

The investigator will notify the accredited METC of the end of the study within a period of 8 weeks. The end of the study is defined as the time point at which all results from the elaborate screening at the central screening facility have become available and responded to. The sponsor will notify the METC immediately of a temporary halt of the study, including the reason of such an action. In case the study is ended prematurely, the sponsor will notify the accredited METC within 15 days, including the reasons for the premature termination. Within one year after the end of the study, the investigator/sponsor will submit a final study report with the results of the study, including any publications/abstracts of the study, to the accredited METC.

11.6 Public disclosure and publication policy

Publication policy is in agreement with the CCMO publication statement. The results of the study will be published in peer-reviewed scientific journals. Both positive and negative results of the study will be disclosed. The principal investigator will always try to publish and/or present results to the general public. Furthermore, the Dutch Kidney Foundation is involved in this project as partner and they will publish a press release about the study.

12. STRUCTURED RISK ANALYSIS

12.1 Potential issues of concern

Given the nature of the PeeSpot device and the ACR | EU urine tests no potential issues of concern regarding risk are foreseen. We refer to the Digitaal Aanschaf Dossier (Appendices ‘D2.1 DAD PeeSpot [versie 1.0, 04-04-2019]’ and ‘D2.2 DAD ACR EU [versie 3.0, 04-04-2019]’) for information regarding the ACR | EU kit and the PeeSpot test and their risk analysis.

13. ADVISORY BOARD

For the current study, an advisory board including individuals with expertise in different areas has been set up. Table 2 presents an overview of the members of the advisory board and their position and interests. We have consulted these individuals multiple times for advice regarding the design and execution of the study. We will also consult them when interpreting the study results.

Name	Position and interest	Institute and department
Prof. Dr. Coen D.A. Stehouwer	Professor of Medicine, vascular medicine	Maastricht UMC, Department of Internal Medicine, Division of Endocrinology
Prof. Dr. Eric Sijbrands	Professor of Medicine, Head of pharmacology, vascular and metabolic diseases section	Erasmus MC, Department of Internal Medicine
Drs. Judith Tjin-A-Ton	General practitioner, kaderhuisarts cardiovascular disease/NHG guidelines	General practice, Amstelveen,
Dr. Wim de Grauw	General practitioner, vascular damage and Chronic Kidney Disease	General practice Berghem and Radboud University Medical Center
Drs. Karen Prantl	Member of Nierpatiënten Vereniging Nederland	Nierpatiënten Vereniging Nederland, Bussum
Dr. R.W. van Etten,	Nephrologist	Dept. Internal Medicine, Amphia Hospital, Breda
Dr. M. Thelen	Clinical chemist	Dept. Clinical Chemistry Amphia Hospital, Breda
Drs. B. Evers	General practitioner Kaderhuisarts Hart- en Vaatziekten	GP Practice Tholos, Zevenbergen
Prof. dr. M.J. Postma	Health economist	Dept. Pharmaco-economy UMC Groningen

Table 2. Overview of the members of the advisory board.

References

1. Coresh J, Byrd-Holt D, Astor BC, Briggs JP, Eggers PW, Lacher DA, Hostetter TH. Chronic kidney disease awareness, prevalence, and trends among U.S. adults, 1999 to 2000. J Am Soc Nephrol 2005;16:180-8.

2. Levin A, Tonelli M, Bonventre J, Coresh J, Donner JA, Fogo AB, Fox CS, Gansevoort RT, Heerspink HJL, Jardine M, et al. Global kidney health 2017 and beyond: a roadmap for closing gaps in care, research, and policy. Lancet 2017;390:1888-917.

3. James MT, Hemmelgarn BR, Tonelli M. Early recognition and prevention of chronic kidney disease. Lancet 2010;375:1296-309.

4. Hillege HL, Janssen WM, Bak AA, Diercks GF, Grobbee DE, Crijns HJ, Van Gilst WH, De Zeeuw D, De Jong PE, Prevend Study Group. Microalbuminuria is common, also in a nondiabetic, nonhypertensive population, and an independent indicator of cardiovascular risk factors and cardiovascular morbidity. J Intern Med 2001;249:519-26.

5. Arnlov J, Evans JC, Meigs JB, Wang TJ, Fox CS, Levy D, Benjamin EJ, D'Agostino RB, Vasan RS. Low-grade albuminuria and incidence of cardiovascular disease events in nonhypertensive and nondiabetic individuals: the Framingham Heart Study. Circulation 2005;112:969-75.

6. Verhave JC, Gansevoort RT, Hillege HL, Bakker SJ, De Zeeuw D, de Jong PE, PREVEND Study Group. An elevated urinary albumin excretion predicts de novo development of renal function impairment in the general population. Kidney Int Suppl 2004;(92):S18-21.

7. Ozyilmaz A, Bakker SJ, de Zeeuw D, de Jong PE, Gansevoort RT, PREVEND Study Group. Selection on albuminuria enhances the efficacy of screening for cardiovascular risk factors. Nephrol Dial Transplant 2010;25:3560-8.

8. Wilson JMG, Jungner G. Principles and practice of screening for disease. Geneva: World Health Organizatin, 1968. no. 34.

9. Atthobari J, Asselbergs FW, Boersma C, de Vries R, Hillege HL, van Gilst WH, Gansevoort RT, de Jong PE, de Jong-van den Berg LT, Postma MJ, et al. Cost-effectiveness of screening for albuminuria with subsequent fosinopril treatment to prevent cardiovascular events: A pharmacoeconomic analysis linked to the prevention of renal and vascular endstage disease (PREVEND) study and the prevention of renal and vascular endstage disease intervention trial (PREVEND IT). Clin Ther 2006;28:432-44.

10. Boersma C, Gansevoort RT, Pechlivanoglou P, Visser ST, van Toly FF, de Jong-van den Berg LT, de Jong PE, Postma MJ, Prevention of Renal and Vascular End Stage Disease Study Group. Screen-and-treat strategies for albuminuria to prevent cardiovascular and renal disease: cost-effectiveness of nationwide and targeted interventions based on analysis of cohort data from the Netherlands. Clin Ther 2010;32:1103-21.

11. Hessels J, Cairo DW, Slettenhaar M, Dogger M. PeeSpot; urine home collection device Innovatieve methode voor verzamelen van portie urine. Ned Tijdschr Klin Chem Labgeneesk 2011;36:249-51.

12. Friedewald WT, Levy RI, Fredrickson DS. Estimation of the concentration of low-density lipoprotein cholesterol in plasma, without use of the preparative ultracentrifuge. Clin Chem 1972;18:499-502.

13. Chaturvedi N. Ethnic differences in cardiovascular disease. Heart 2003;89:681-6.

14. van den Belt SM, Gracchi V, de Zeeuw D, Heerspink HJ. Comparison of urine collection methods for albuminuria assessment in young children. Clin Chim Acta 2016;458:120-3.

15. Brinkman JW, de Zeeuw D, Duker JJ, Gansevoort RT, Kema IP, Hillege HL, de Jong PE, Bakker SJ. Falsely low urinary albumin concentrations after prolonged frozen storage of urine samples. Clin Chem 2005;51:2181-3.

16. Kidney Disease Impoving Global Outcomes (KDIGO). KDIGO Clinical Practice Guideline for the Management of Blood Pressure in Chronic Kidney Disease. 5th ed. Kidney Int Suppl, 2012. no. 2.

17. Levey AS, Stevens LA, Schmid CH, Zhang YL, Castro AF,3rd, Feldman HI, Kusek JW, Eggers P, Van Lente F, Greene T, et al. A new equation to estimate glomerular filtration rate. Ann Intern Med 2009;150:604-12.

18. Levey AS, de Jong PE, Coresh J, El Nahas M, Astor BC, Matsushita K, Gansevoort RT, Kasiske BL, Eckardt KU. The definition, classification, and prognosis of chronic kidney disease: a KDIGO Controversies Conference report. Kidney Int 2011;80:17-28.

19, Gansevoort RT, Verhave JC, Hillege HL,, Burgerhof JGM, Bakker SLJ, de Zeeuw D, de Jong PE. The validity of screening based on spot morning urine samples to detect subjects with icroalbuminuria in the general population. Kidney Int 2005:94:S28-S35.
